# Teaching critical thinking about health information and choices in secondary schools: human-centred design of digital resources

**DOI:** 10.12688/f1000research.132580.1

**Published:** 2023-05-11

**Authors:** Sarah Rosenbaum, Jenny Moberg, Faith Chesire, Michael Mugisha, Ronald Ssenyonga, Marlyn A Ochieng, Clarisse Marie Claudine Simbi, Esther Nakyejwe, Benson Ngatia, Gabriel Rada, Juan Vásquez-Laval, José Damián Garrido, Grace Baguma, Sam Kuloba, Edward Sebukyu, Richard Kabanda, Irene Mwenyango, Tonny Muzaale, Pamela Nandi, Jane Njue, Cyril Oyuga, Florian Rutiyomba, Felecien Rugengamanzi, Joan Murungi, Allen Nsangi, Daniel Semakula, Margaret Kaseje, Nelson Sewankambo, Laetitia Nyirazinyoye, Simon Lewin, Andrew D Oxman, Matt Oxman

**Affiliations:** 1 Centre for Epidemic Interventions Research, Norwegian Institute of Public Health, Oslo, 0213, Norway; 2 Tropical Institute of Community Health and Development in Africa, Kisumu, Kenya; 3 Institute of Health and Society, Faculty of Medicine, Universitetet i Oslo, Oslo, Oslo, Norway; 4 School of Public Health, College of Medicine and Health Sciences, University of Rwanda, Butare, Southern Province, Rwanda; 5 School of Medicine, College of Health Sciences, Makerere University, Kampala, Central Region, Uganda; 6 Epistemonikos Foundation, Santiago, Santiago Metropolitan Region, Chile; 7 National Curriculum Development Centre, Kampala, Uganda; 8 Ministry of Education and Sports, Kampala, Uganda; 9 Faculty of Health Sciences, Uganda Martyrs University, Kampala, Central Region, Uganda; 10 Ministry of Health, Kampala, Uganda; 11 Uganda National Examinations Board, Kampala, Uganda; 12 Huma Girls Secondary School, Kisumu, Kenya; 13 Kenya Institute of Curriculum Development, Nairobi, Kenya; 14 Rwanda Basic Education Board, Kigali, Rwanda; 15 Health Systems Research Unit, South African Medical Research Council, Cape Town, South Africa; 16 Department of Health Sciences, Norwegian University of Science and Technology (NTNU), Ålesund, Norway; 17 Faculty of Health Sciences, Oslo Metropolitan University, Oslo, Norway

**Keywords:** critical thinking, critical health literacy, informed decision making, infodemic, health education, secondary school, educational design, human centred design

## Abstract

Background

Learning to thinking critically about health information and choices can protect people from unnecessary suffering, harm, and resource waste. Earlier work revealed that children can learn these skills, but printing costs and curricula compatibility remain important barriers to school implementation. We aimed to develop a set of digital learning resources for students to think critically about health that were suitable for use in Kenyan, Rwandan, and Ugandan secondary schools.

Methods

We conducted work in two phases collaborating with teachers, students, schools, and national curriculum development offices using a human-centred design approach. First, we conducted context analyses and an overview of teaching strategies, prioritised content and collected examples. Next, we developed lessons and guidance iteratively, informed by data from user-testing, individual and group interviews, and school pilots.

Results

Final resources include online lesson plans, teachers’ guide, and extra resources, with lesson plans in two modes, for use in a classroom equipped with a blackboard/ﬂip-chart and a projector. The resources are accessible offline for use when electricity or Internet is lacking. Teachers preferred the projector mode, as it provided structure and a focal point for class attention. Feedback was largely positive, with teachers and students appreciating the learning and experiencing it as relevant. Four main challenges included time to teach lessons; incorrect comprehension; identifying suitable examples; and technical, logistical, and behavioural challenges with a student-computer mode that we piloted. We resolved challenges by simplifying and combining lessons; increasing opportunities for review and assessment; developing teacher training materials, creating a searchable set of examples; and deactivating the student-computer mode.

Conclusion

Using a human-centred design approach, we created digital resources for teaching secondary school students to think critically about health actions and for training teachers. Be smart about your health resources are open access and can be translated or adapted to other settings.

## Introduction

Claims about how to care for our health are everywhere, spread by friends, family, news media, industry, healthcare professionals, policymakers, researchers, and others. Many of these claims unreliable, ^1^ but people often lack the skills needed to assess them. ^2^^,^^3^ When we believe unreliable claims, we might take ineffective or harmful actions, or fail to take helpful actions. The Covid-19 pandemic showed how easily unreliable claims ^4^^–^^6^ and research ^7^^–^^9^ spread, impacting public trust and protective behaviours. ^10^^,^^11^

Publicly debunking untrustworthy information has value, but the effectiveness of this retroactive strategy might be limited. Misinformation, once spread, can be resistant to correction; an alternative narrative may not exist (for instance, there may not be a safe or proven alternative treatment); and individuals may reject the scientific process or the source altogether rather than change their established views about some topics. ^12^ Pre-emptively ‘inoculating’ people against misinformation ^13^^,^^14^ by teaching them to think critically about claims of what works, and how to make informed decisions, has the potential to provide broader, more long-lasting protection.

In earlier work developing and evaluating Informed Health Choices (IHC) resources for primary school students, we have shown that it is feasible to teach critical thinking skills about health actions to children as young as 10-years old. ^15^^–^^20^ In that project, we created a framework of principles that are important for people to understand when assessing the reliability of healthcare claims and making informed choices: the IHC Key Concepts. ^21^^,^^22^ This framework is a starting point for designing curricula, learning resources, and evaluation tools. Focusing on a selection of the IHC Key Concepts, we then developed printed learning resources for primary school children (age 10-12 years) and their teachers ^15^ and a podcast for parents. ^16^

To evaluate the effect of these resources, we conducted two randomised trials in Uganda, one including 120 schools and more than 10,000 children, and another that included 675 parents and guardians of primary school children. ^23^ The primary outcome measure was responses to multiple-choice questions that measure respondents’ ability to apply IHC Key Concepts: the Claim Evaluation Tools. ^17^^,^^18^ The primary school trial demonstrated that use of the resources led to a large improvement in the ability of children and teachers to apply IHC Key Concepts to hypothetical scenarios. ^17^ The podcast trial showed a smaller, similar effect among parents. ^18^ Follow-up data showed that children retained their learning for at least one year, ^19^ while the performance of the parents declined. ^20^ The primary school resources have been translated into 12 languages and adapted for use in other countries. ^24^

Alongside the trials, we undertook process evaluations to explore barriers and facilitators for scaling up use of the learning resources, potential adverse effects, and potential additional benefits. ^25^^,^^26^ We found that children, teachers, parents, and district education officers valued the IHC primary school resources. The human-centred design approach we employed - iteratively addressing user and stakeholder concerns prior to and during development - was likely an important contributing factor for this positive reception. However, the study pointed to two important implementation barriers in Uganda: printing costs and lack of time in the school schedule.

Informed by these findings, we began the current five-year project in 2019 to develop and evaluate resources for lower secondary schools (age 13-16) in Kenya, Rwanda, and Uganda. Since drafting this article, we have evaluated the resources in three parallel randomised trials, ^27^^–^^29^ are carrying out process evaluations, ^30^^–^^32^ and will conduct one-year follow-up trials in each of the three countries.

This article describes the development of these resources that took place prior to evaluation during the first 2.5 years of the project in two phases. We began development with this set of objectives (see protocol ^33^):

To explore how we might develop resources that are

• Digital (avoiding printing costs)
• Suitable for use with schools’ available digital technology and infrastructure
• Compatible with national curricula
• Based on evidence about effective strategies for teaching critical thinking
• Experienced as accessible, useful, usable, understandable, credible, and desirable by students and teachers, and well-suited to use in their schools
• Easily translatable and adaptable to other contexts
• Sustainable (i.e., not dependent on our team for rolling out at scale)

We organised the work in two phases: 1) a set of preliminary studies (involving data documented and discussed elsewhere) and 2) an iterative development phase with data collection and analysis reported in this article. Although the preliminary studies have already been published elsewhere, findings from those studies resulted in reframing of some of our objectives and provided the basis for content and design decisions made in the second phase. Therefore, we describe methods and results from both phases (with references for more detail about phase one). In the discussion, we describe how this study may inform development of similar educational resources for teaching critical thinking.

## Methods

We employed perspectives and methods from human-centred design. This is an iterative approach to creating products, systems and services that places users’ and other stakeholders’ needs and experiences at the centre of the design process. ^34^^,^^35^ Early and continued engagement with stakeholder groups and multidisciplinary collaboration are central components of a human-centred design approach. ^36^^,^^37^ We employed qualitative data collection and analysis methods to explore the user experiences of multiple types of stakeholders for the purpose of informing the development process.

We established a core team with backgrounds in health systems and public health research, design, journalism, education, social science, statistics, and information and communication technology (ICT). Research leadership was shared among senior team members in East Africa and Norway. Three PhD fellows (one female and two male) based in Kisumu (Kenya), Kigali (Rwanda) and Kampala (Uganda), engaged with stakeholders and collected and analysed data, supported by their local teams including researchers with experience developing and evaluating the IHC primary school resources. All teams contributed to design and content development, led by the team based in Norway who also analysed data. The ICT team, based in Chile, developed the technical solutions.

**Figure 1.**
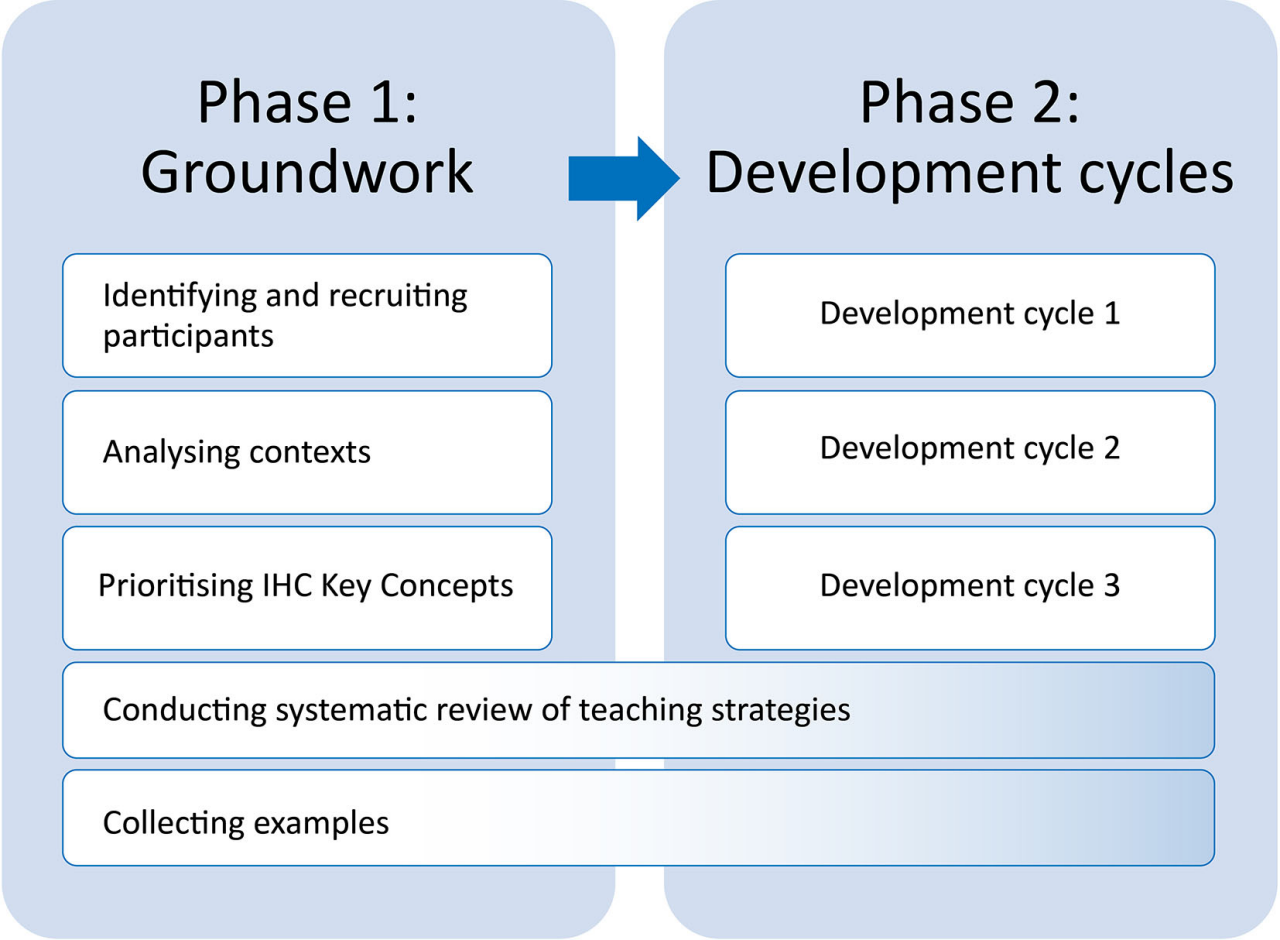
Phases of the work.

We conducted this work in two phases: 1) Groundwork and 2) Development cycles ( Figure 1). The first phase began in August 2019, the second phase began January 2020 and ended in April 2022.

### Identifying and recruiting participating stakeholders

We defined stakeholders as people or organisations that have a vested interest in the process or results of the study. Intended users – secondary school teachers and students – were the key stakeholders. Additional stakeholders included curriculum developers and other education policymakers, school administrators, parents, educational researchers, researchers in public or clinical health, and health professionals.

For the purpose of providing continuous input, we established groups of stakeholders in each country early in the project. We formed teacher networks, recruiting *via* formal invitation letters to teachers who worked in a mix of government funded and private schools, with varied ICT resources (Uganda), and in rural, semi-rural, and urban environments (Kenya and Rwanda). We formed student networks by asking members of the teacher networks to suggest lower secondary school students who were interested, likely to contribute, and able to participate without adversely affecting their schoolwork, sending them formal invitations and assent forms, and consent forms to parents. Due to Covid-related school closures in parts of Development Cycle 1, we also established “home networks” of students that we had access to when schools were closed. Home networks included both students that were already recruited to the student network and additional students whom team members could easily reach from nearby communities. We employed convenience sampling and used the same assent forms and consent forms to parents as for the student networks.

We engaged with the national curriculum development offices to establish channels of contact and collaboration. We formed national advisory panels of policy makers at the ministerial and regional and district levels, school directors, head teachers, leaders of teacher unions, and representatives of parents’ groups and civil society. We created an international advisory panel of people from 18 countries who had expertise in education, education policy, and relevant areas of research, such as health literacy, evidence-informed decision making, science communication and ICT. Additionally, during the final cycle of development, we recruited seven schools across the three countries to pilot use of the resources in one or more classes over a school semester.

Apart from people in the international advisory panel, many of whom were a part of our existing professional networks, and some of the students in home networks, we did not have established relationships with the stakeholders prior to the onset of this project.

Details of how we selected and recruited each stakeholder group and characteristics of participants and schools are described in Tables 1- 3. More information about the methods, degrees, and nature of engagement with different stakeholders, can be found in a protocol for evaluating stakeholder engagement. ^38^

**Table 1.** Participant selection and recruitment methods.

Stakeholder group	Country	What/who we sought	Sampling/selection	Method of approach
**Teacher networks**	Kenya, Rwanda, Uganda	*Schools:* mix of government-funded/private and rural/semi-urban/urban *Teachers:* diverse (gender, level of education, years of experience, subject areas)	Stratified sampling used to select schools, based on school ownership and ICT resources. We contacted head teachers and asked them to suggest one or more teachers to be a part of the network	We sent formal invitation letters to teachers
**Student networks (including home networks)**	Kenya, Rwanda, Uganda	Lower secondary school students who were: ‐ interested ‐ likely to contribute ‐ able to participate without adversely affecting their schoolwork	We asked each member of the teacher networks to suggest one or more students to join student networks in each country, and aimed for a mix of top, average and low academic performers, as well as a mix of gender and age	We sent formal invitations to the students and their parents, with consent forms for the parents and assent forms for the students to sign. In some cases, we visited parents at the homes to deliver the letters and explain the study
**National curriculum developers**		Members of the national curriculum committees		We invited them via email, telephone, or face-to-face visits
**National advisory panels**	Kenya, Rwanda, Uganda	Policymakers at the ministerial and regional and district levels including curriculum developers, school directors, head teachers, leaders of teacher unions, and representatives of parents’ groups and civil society	We identified potential members by consulting informal contacts, the teacher networks, and initial invitees (snowballing)	We invited them via email, telephone, or face-to-face visits
**International advisory panel**	Australia, Brazil, Canada, China, Croatia, Iran, Ireland, Italy, Kenya, Mexico, Norway, Palestine, Rwanda, South Africa, Spain, Uganda, UK, USA, 3ie (international)	People from low-, middle-, and high-income countries with expertise in education and education policy, relevant areas of research (e.g., education, health literacy, science communication, evidence-based practice, critical thinking), design, ICT, and learning games	We identified these through existing contacts, publications, and snowballing	We sent invitations via email
**Pilot schools**	Kenya	Mix of schools by ownership and geographical: two semi urban public schools one rural public school	Stratified sampling to select schools by ownership and location. We contacted head teachers and asked them to suggest one or more teachers to be a part of the study	We formally introduced the study objectives to ministry of education officials at national, regional and county in Kenya. Together with the officials selected based on selected by ownership and geographical location and contacted the head teachers via formal invitation letters asking their schools to participate in the study
	Rwanda	Two Schools varied from location (urban/rural), had ICT facilities and were able to dedicate their time in the pilot of learning resources	Purposively selected them with recommendation from Rwanda Education Board (REB)	We sent a request to REB and REB requested two schools in a letter addressed to school directors
	Uganda	Two privately owned schools (rural/urban)	Selected schools in proximity to the research teams. We contacted head teachers and asked them to suggest one or more teachers to participate in the pilot. Then we enrolled the teacher after consenting them	We sent formal invitation letters to schools’ administration (head teachers)

**Table 2.** Participant characteristics.

Stakeholder group	Country	# people	More detail
**Teacher networks**	Kenya	22 teachers	*Schools:* 4 urban, 6 semi-urban, 12 rural *Subjects taught:* Business studies, Chemistry, History, Biology, Kiswahili, French, Mathematics, Home-science, CRE, English *Gender:* 12 female, 11 male
	Rwanda	19 teachers	*Schools:* 10 schools (1 private, 5 public and 4 government aided schools). *Teaching experience:* 4 to 10 years. *Subjects taught:* 13 Teachers teach biology. Others teach Chemistry, Physics, Math, French and English. *Gender:* 7 female, 12 male
	Uganda	22 teachers	*Schools:* 5 public schools with at least a projector, 5 private schools with at least a projector, 6 public schools with no projector and 6 private schools with no projector. *Gender:* 7 female, 15 male Teaching experience: range was 2-34 years, average of 12 years *Subjects taught*: 7 biology & Chemistry, 7 Maths & Physics, 3 geography & History, 4 English & ICT, 1 Home science
**Student networks**	Kenya	24 students	*Schools:* 1 public urban, 1 public rural and 1 private urban. *Students:* 15–18-year-olds, (18 female, 6 male)
	Rwanda	19 students	*Schools:* 5 public schools, 4 private schools. *Students:* 10–16-year-olds *Gender:* 10 female, 9 male
	Uganda	21 students	*Schools:* 1 public with at least a projector, 1 private with at least a projector and 1 private with no projector. *Ages:* 14–19-year-olds *Gender:* 13 female, 8 male
**National curriculum developer office**	Kenya	1 institute	Kenya Institute of Curriculum Development (KICD)
	Rwanda	1 institute	Rwanda Basic Education Board (REB)
	Uganda	1 institute	National Curriculum Development Centre (NCDC)
**National advisory groups**	Kenya	12	Researchers and practitioners from various fields (education, health, design/IT), curriculum specialists and teachers)
	Rwanda	11	1 curriculum developer, 1 ICT specialist, 3 from health sector (adolescent, community health and health services), 1 educational researcher and 5 educational development partners working in Rwanda
	Uganda	14	Curriculum developers, Policy makers (commissioners, district education officers) from government, school head teachers, teachers, education researchers
**International advisory group**	18 countries: Australia, Brazil, Canada, China, Croatia, Iran, Ireland, Italy, Kenya, Mexico, Norway, Palestine, Rwanda, South Africa, Spain, Uganda, UK, USA	56 people	Researchers and practitioners from various fields: education, public and clinical health, health literacy, design/IT/games, methodologists, and funders/international organisations

**Table 3.** Characteristics of schools participating in pilot of ten lessons.

Country	Number and type of schools	# of classes/groups	Class level	Total # of students
Kenya	2 public semi urban and 1 public- rural	5 classes	Form one (Students that are equivalent in age to Rwanda and Uganda “senior two in ordinary level”, approx. 13-15 years old)	Approximately 200 students
Rwanda	1 public/rural/and day school and 1 government aided/urban/and day school	2 classes	Senior two in ordinary level	84 students
Uganda	2 private schools (one with a projector and the other without)	2 groups (conducted on school premises during a period of school closure due to Covid)	Senior two in ordinary level	23 students

### Phase 1: Groundwork

The groundwork phase consisted of a series of preliminary studies. More detail about the methods and results can be found in separate publications (see overview in Table 4).

**Table 4.** Overview of preliminary studies.

Study aim	Methods/Study design	Data sources	More detail
**Analysing contexts in Kenya, Rwanda, and Uganda**	Individual and group interviews, document analysis, non-participatory observation	*Interviews:* Teachers, students, curriculum developers, ICT support staff *Document analysis:* learning materials for health and science subjects, curriculum documents, ICT policy documents *Observation*: School classes	^39^^–^^41^
**Prioritising IHC Key Concepts (learning goals)**	Iterative, structured consensus process	Curriculum specialists, teachers, and researchers	^54^
**Identifying teaching strategies for critical thinking**	Overview of systematic reviews	Systematic reviews of the effects of teaching strategies for critical thinking	^44^
**Collecting examples**	Individual and group interviews	Secondary school students in Kenya, Rwanda, and Uganda	^45^

**Analysing contexts**

To inform the development of the resources, as well as their potential implementation in Kenya, Rwanda and Uganda lower secondary schools, we explored if there was demand for learning resources to teach critical thinking; if such resources were in use; how the content maps onto existing curricula; what administrative approval was necessary; what ICT infrastructure is available in schools and how it is used in schools. We analysed the three country contexts. Detailed methods are reported in a separate publication. ^39^^–^^41^

To synthesize findings from these studies in a way that was useful for developing educational resources, we drew on the the behaviour change wheel. ^42^ This is a framework for characterising and designing interventions for behaviour change, built around three essential conditions for change: Capability, Opportunity, and Motivation (the COM-B system). We organised findings from the three context analyses according to these three themes. The synthesized findings from these three context analyses supplemented our original set of development aims and provided a much more detailed understanding of contextual challenges before we started the development phase.

**Prioritising IHC Key Concepts**

Due to the scope of our research funding, as well as the pandemic and school closures, we could not pilot and evaluate resources that took longer to use than one school semester. To prioritise which of the 49 IHC Key Concepts to include in the learning goals for the resources, we organised a structured, iterative consensus process with curriculum specialists, teachers, and researchers. Detailed methods are reported in a separate publication. ^43^

**Identifying teaching strategies**

To identify effective, relevant teaching strategies to inform the resources, we conducted an overview of systematic reviews of strategies for teaching critical thinking. Detailed methods are reported in a separate protocol. ^44^

**Collecting examples**

To identify relevant and engaging examples for the resources, we interviewed Kenyan, Rwandan, and Ugandan secondary school students (aged 14 to 16) from the student networks, via WhatsApp. We collected examples of health actions, their claimed and actual effects, and information sources. The students rated their interest in a broad range of health actions and conditions, and we used those with higher scores as examples in the resources. Detailed methods are reported in an online report. ^45^

### Phase 2: Development cycles

**Figure 2.**
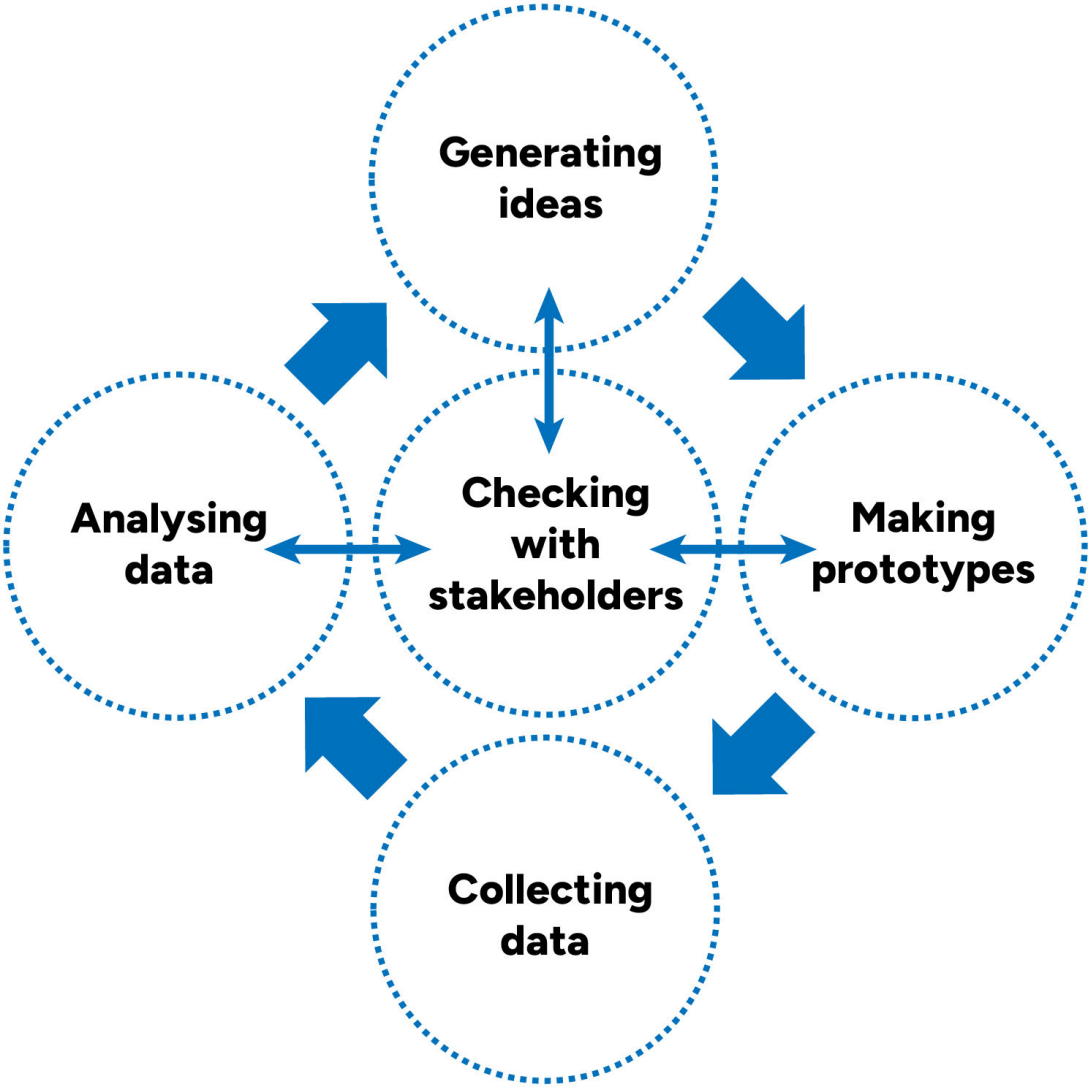
Single development cycle.

Starting with what we learned and developed in the preliminary studies, we developed resources in three iterative cycles of content creation and feedback. Each development cycle ( Figure 2) included:

- Generating ideas
- Prototyping
- Pilot testing prototypes and collecting data
- Analysing problems
- Checking our analysis of the most important problems and solution ideas with key stakeholders, drawing on the concept of “member checking” ^46^

We also sought *ad hoc* feedback from the student and teacher networks as needed.

**Generating ideas**

We generated content and design ideas in two ways: brainstorming ^33^ internally, within the team; and identifying ideas suggested in external feedback, from participating stakeholders. ^47^ We selected ideas based on considerations such as consistency with what teachers and students said they valued, and ease of implementation.

**Prototyping**

A prototype is a sketch or model. We developed increasingly refined prototypes in each cycle. We used the platforms Google Drive (including Google Docs and Google Slides) and Adobe XD for drafting, editing, and sketching. We drafted and edited text using Google Docs, exported early prototypes to PDF format, and used Google Slides and Adobe XD later to create interactive sketches. Final versions were published as web pages, using Google Drive as a content editing platform.

**Testing prototypes and collecting data**

The three PhD fellows conducted interviews in their respective settings, supported by their research assistants. They had university degrees (Bachelor of Statistics, Master of Epidemiology & Biostatistics, Master of Public Health, and Master of Community Health and Development) and have attended PhD-level courses in qualitative study methods.

***Individual interviews employing user testing methods***

Using semi-structured guides ^48^ that were pilot tested, the three PhD fellows conducted user test with individual teachers and students, to explore how they experienced prototypes and how we might make improvements. Data were collected face to face or online when covid measures restricted face to face meetings. When possible, user testing immediately followed a pilot lesson, so the participant had their experiences from the lesson fresh in mind. Interview duration was approximately one hour. Occasionally, teams printed the digital prototypes on paper to collect feedback where there was no access to a device or internet connection.

Each user testing session had three main parts. First, interviewers introduced themselves and the reasons for the interview. Next, they employed a think-aloud approach: a method of qualitative data collection that encourages the participant to articulate their thoughts while performing a task, ^49^^–^^51^ in this case reviewing prototypes of the resources. The last part was conducted as a semi-structured interview.

We captured data as observers’ notes and audio recordings. We designed the interview guides to explore different facets of the user experience, including usefulness, understandability, usability, credibility, desirability, and identification, based on a revised version of Morville’s framework of user experience ( Table 5). ^34^

**Table 5.** Revised version of Morville’s framework of user experience.

Facet	Description
Usefulness	Does this product have practical value for the user?
Usability	How easy and satisfying is this product to use?
Understandability	Does the user recognize what the product is, and do they understand the content? (Subjective experience of understanding)
Credibility	Is the content trustworthy?
Desirability	Is this product something the user wants – has a positive emotional response to?
Identification	Does the user feel the product is for”someone like me” or is it alienating/foreign-feeling? (e.g., age, gender, culture–appropriate)

***Group interviews***

The PhD fellows conducted semi-structured interviews with groups of students or teachers to explore their experiences of the prototypes, using pilot-tested interview guides. ^48^ When possible, interviews were scheduled immediately after a pilot lesson when the participants had their experiences fresh in mind. Sessions lasted approximately one hour.

We chose group interviews, rather than user testing or individual interviews, when we anticipated that the former would improve the quality of the data, for instance by increasing students’ confidence in speaking with researchers. Group interviews took place at schools or other locations determined to be practical for the participants. After introducing themselves and explaining reasons for the interview, one researcher moderated and one or more researchers observed and took notes. Sometimes, teachers were present during student interviews. With written informed consent (from parents and teachers) and assent (from students), we audio-recorded sessions.

***Piloting and observation***

We facilitated pilots of the prototypes to explore how teachers and students used and experienced them in as natural settings as possible. Early pilots involved single lessons, with one of the PhD fellows sometimes assuming the role of the teacher. These took place both at schools and in other settings, such as students’ neighbourhoods, when schools were closed due to the pandemic. The final set of pilots included a full set of lessons taught by teachers at schools, over a school term. (In Uganda, although schools were still closed, we got permission for students from the student network and teachers to meet at the schools for the purpose of conducting pilot lessons.) PhD fellows or research assistants observed and took notes using a structured guide, ^48^ without intervening. We followed up pilots with either individual or group interviews.

***“Critical Thinking about Health” Test***

After piloting a full set of lessons, students took the “Critical Thinking about Health” Test. ^52^ Administering the test had two purposes: validating the items included in the test and giving us a sense of whether the prototypes had the intended effects. We report the validation in detail in a separate article. ^52^

***Consulting the advisory groups***

Twice annually, we emailed the international advisory group, to update them on the project, and ask for feedback on specific parts or prototypes. We entered their feedback in a spreadsheet, ^47^ familiarised ourselves with those data, and discussed and agreed on how to deal with any specific suggestions. We held face-to-face meetings with the national advisory groups to keep them updated and seek feedback related to ensuring the sustainability and future scaling up of resources, if shown to be effective.

**Data analysis**

We transcribed and translated several of the audio recordings from individual and group interviews. The three PhD fellows and their three research assistants reviewed transcriptions, their own interview and observation notes, and recordings, and extracted data about negative and positive experiences. They entered the data into Excel spreadsheets, ^47^ supplemented with quotations where relevant, and tagged the data using pre-determined codes related to the nature of an experience (e.g., “negative”); the “location” of the experience, i.e., the relevant part of the resources (e.g., “illustration/graphics”), and suggested implication (e.g., “consider changes”). We did not anticipate differences in results between male and female students, so coding did not include gender identification. The prototyping team reviewed the coded data, flagged data entries that they did not understand, suggested changes to codes, and discussed the suggestions with the PhD fellows and their research assistants.

After agreeing with the original coders on the final codes, the three-person prototyping team sorted data entries according to the nature, implication and location, and re-reviewed them, focusing on data tagged with the implication “showstopper” or “consider changes”. New topic codes emerged during this process and were added as thematic labels of issues that needed addressing ( *e.g.,* “Time”, “Conceptual misunderstanding”, “student-computer mode”). This coding scheme evolved throughout. For an example from the dataset, ^47^ see the “Topics” column in the Development Cycle 2 – codes and response options. When data analysis raised important unanswered questions or resulted disagreement about interpretation, the team either contacted teacher or student networks for additional input, or adjusted interview guides to include these issues in the next development cycle.

The prototyping team drafted a description and assessment of the most important problems with the prototypes, with an initial set of ideas for solutions. The whole project team discussed these drafts, reached a consensus about which were the most important problems and brainstormed additional ideas for solutions. Based on this input, the prototyping team made the final decision about which solutions to implement.

**Checking with stakeholders**

Drawing on the method “member checking”, ^46^ we carried out “stakeholder checking”, checking our analysis and ideas with the teacher networks and curriculum developers. At the end of each development cycle, we presented descriptions of what we considered the most important problems and our proposed solutions. We asked about the accuracy of the analysis, their reactions to the proposed solutions, and any suggestions of their own. We modified solutions based on this input.

***Ad hoc* feedback**

At different points within each development cycle, we also contacted people in the teacher and student networks for quick feedback on specific issues, for instance what to call the resources.

**Figure 3.**
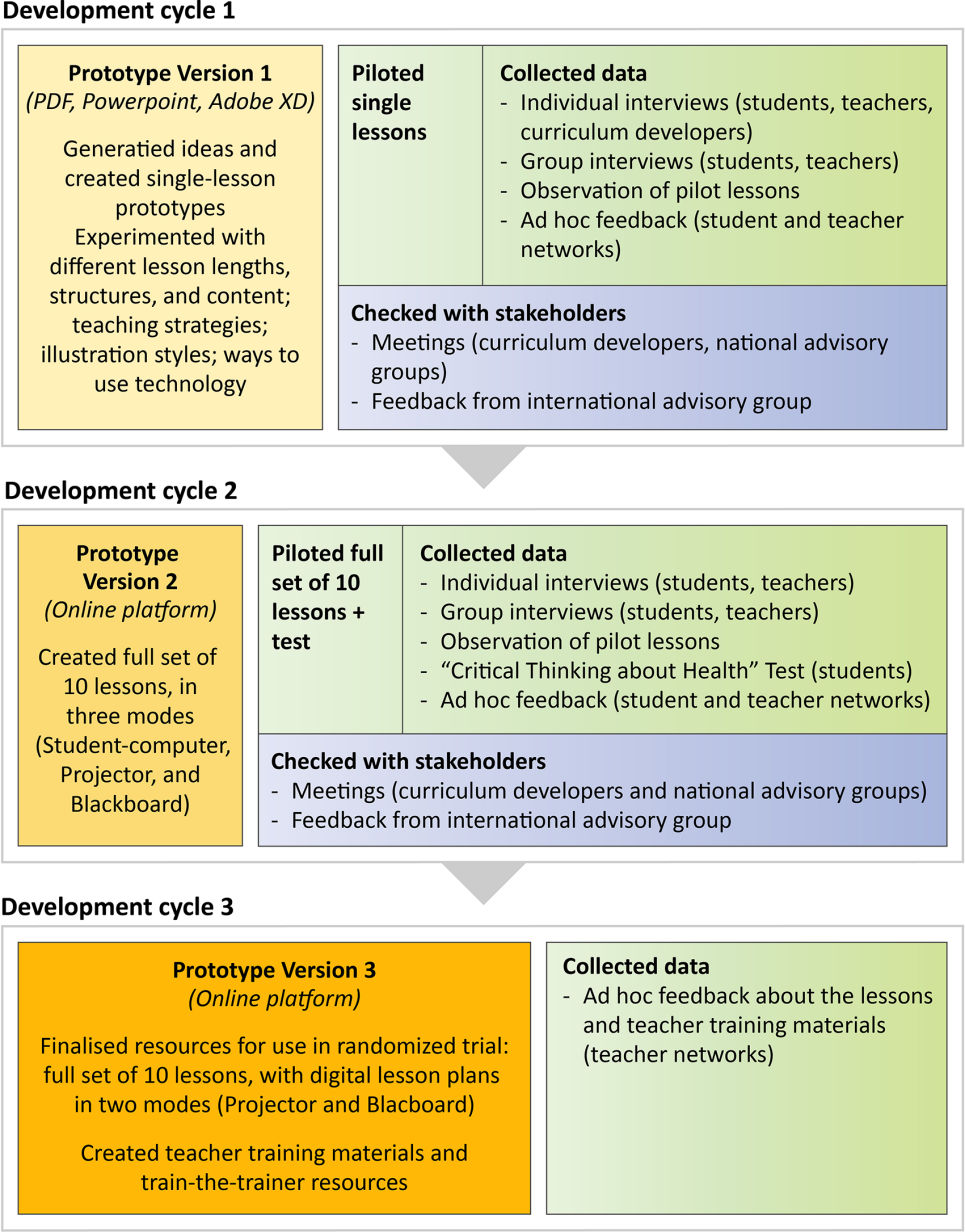
Overview of the three development cycles.

Figure 3 and Table 6 provide an overview of what we did in the three development cycles.

**Table 6.** Scope of teacher and student data collection (interviews, lesson pilots, tests).

Method	Development cycle 1	Development cycle 2
**Student interviews**	**18 interviews** (11 individual user tests and 7 group interviews)	**17 interviews** (2 individual user tests and 15 group interviews)
**Teacher interviews**	**21 interviews** (16 individual user tests and 5 group interviews)	**35 interviews** (33 individual user test interviews and 2 group interviews)
**Piloting lessons and test**	**Our team observed 4 pilot lessons**	**9 classes piloted the full set of 10 lessons**
		**Our team observed 37 pilot lessons**
		**259 students** took “Critical Thinking about Health” test

### Ethics approval

We obtained ethics approval for the entire project from local institutional review boards and national research governing councils in Kenya, Rwanda, and Uganda: the Rwanda National Ethics Committee (approval number 916/RNEC/2019); Masinde Muliro University of Science and Technology Institutional Ethics Review Committee and the Kenyan National Commission for Science, Technology and Innovation (approval number NACOSTI/P119/1986); and Makerere University School of Medicine Research Ethics Committee (REC REF 2020-139) and Uganda National Council of Science and Technology (HS916ES).

## Results

We present two different types of results that emerged from this work: the designed output (final version of the resources), and the knowledge output that informed our development (findings from Phase 1 and 2). We report the designed output first, to give the reader an overview of the resource features and components we refer to in the findings.

### Designed output: the final version of “Be smart about your health” resources

**Figure 4.**
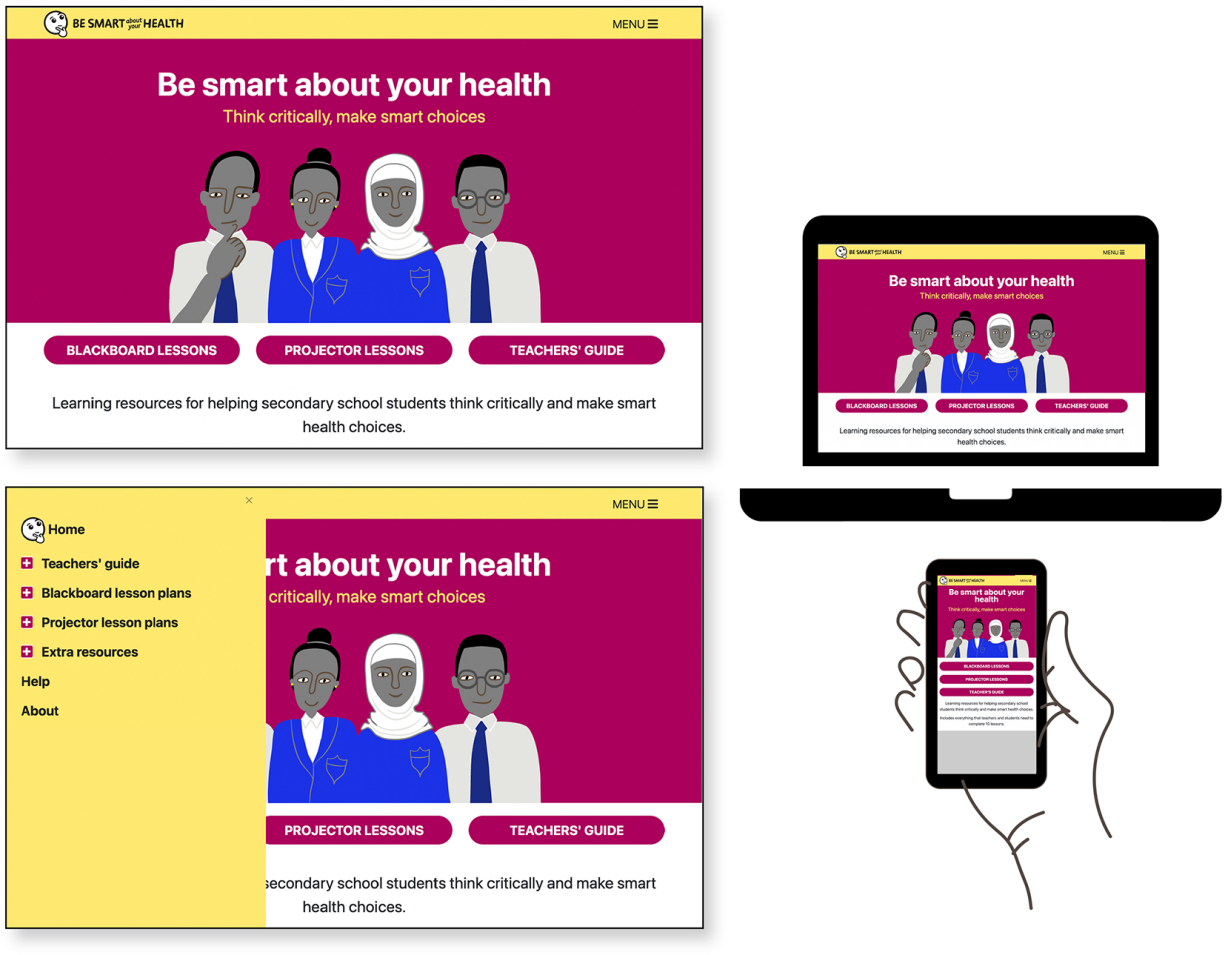
Final version of the “Be smart about your health” resources. Contents include *Teachers’ guide*, *Blackboard lesson plans, Projector lesson plans*, and *Extra resources.*

**Figure 5.**
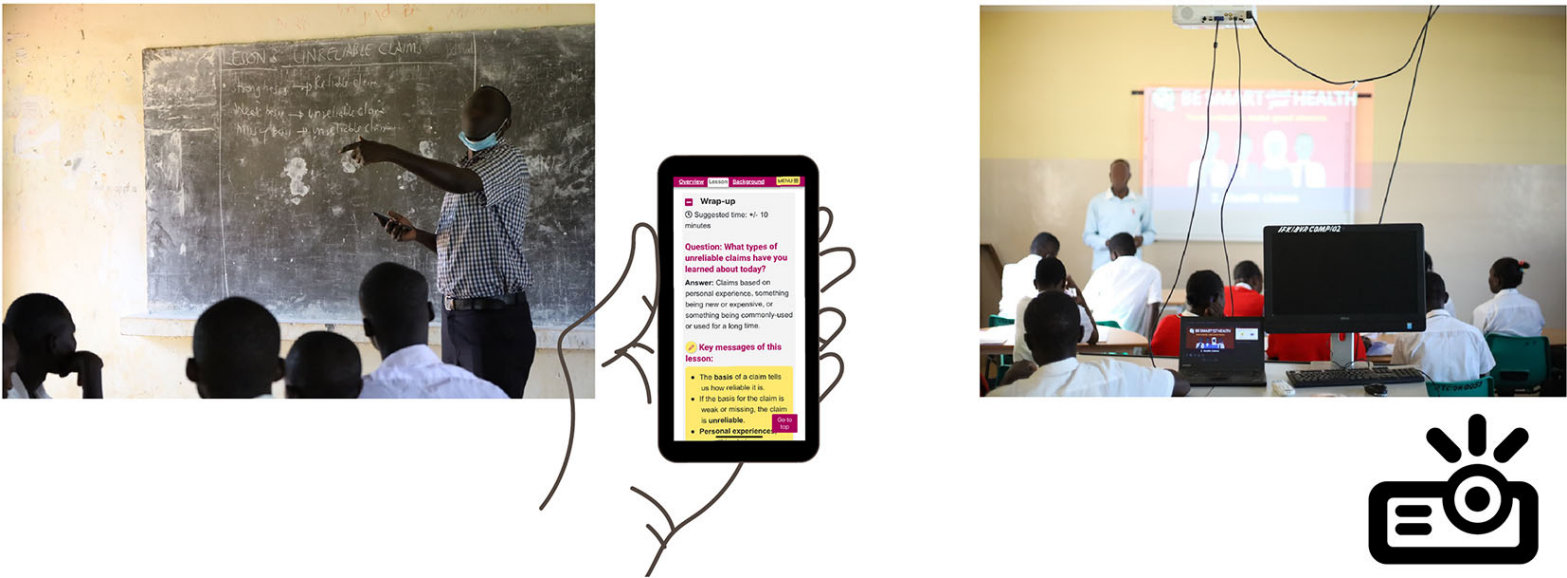
Lessons designed in two modes: *Blackboard mode* for use in classrooms equipped with a blackboard or a flipboard, and *Projector mode* for use in classrooms with a projector.

**Figure 6.**
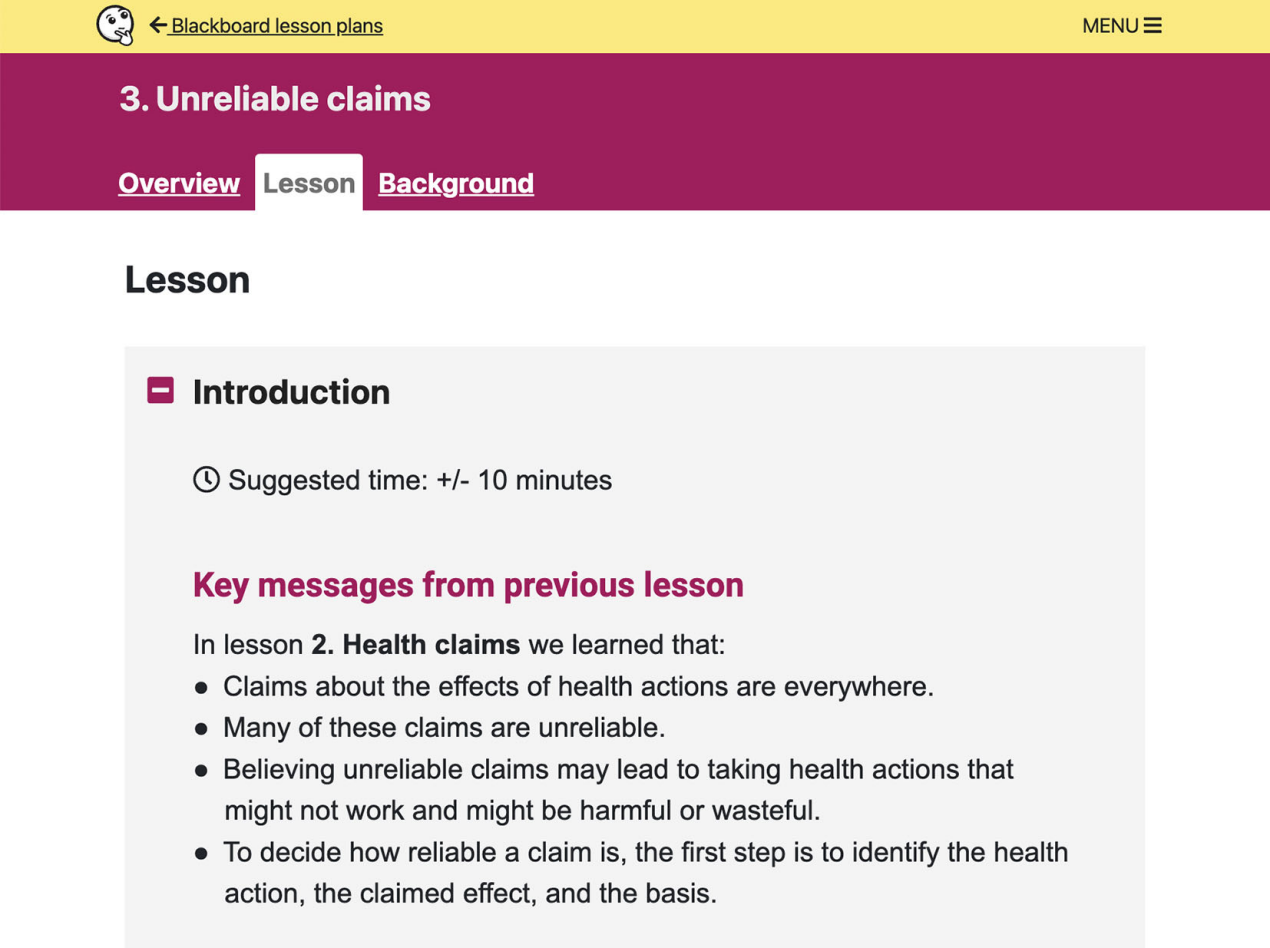
Lesson plan content. Each lesson plan includes an *Overview*, *Lesson*, and *Background* section for teachers. Each *Lesson* has three parts: Introduction, Activity, and Wrap-up.

**Figure 7.**
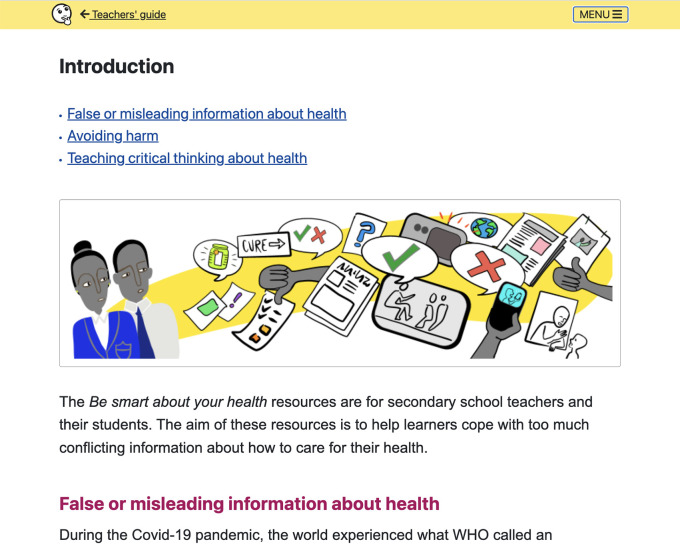
Teachers’ guide includes: Introduction, Overview of the lessons, Using the resources, Background for teachers, Development and evaluation, and Other relevant resources.

**Figure 8.**
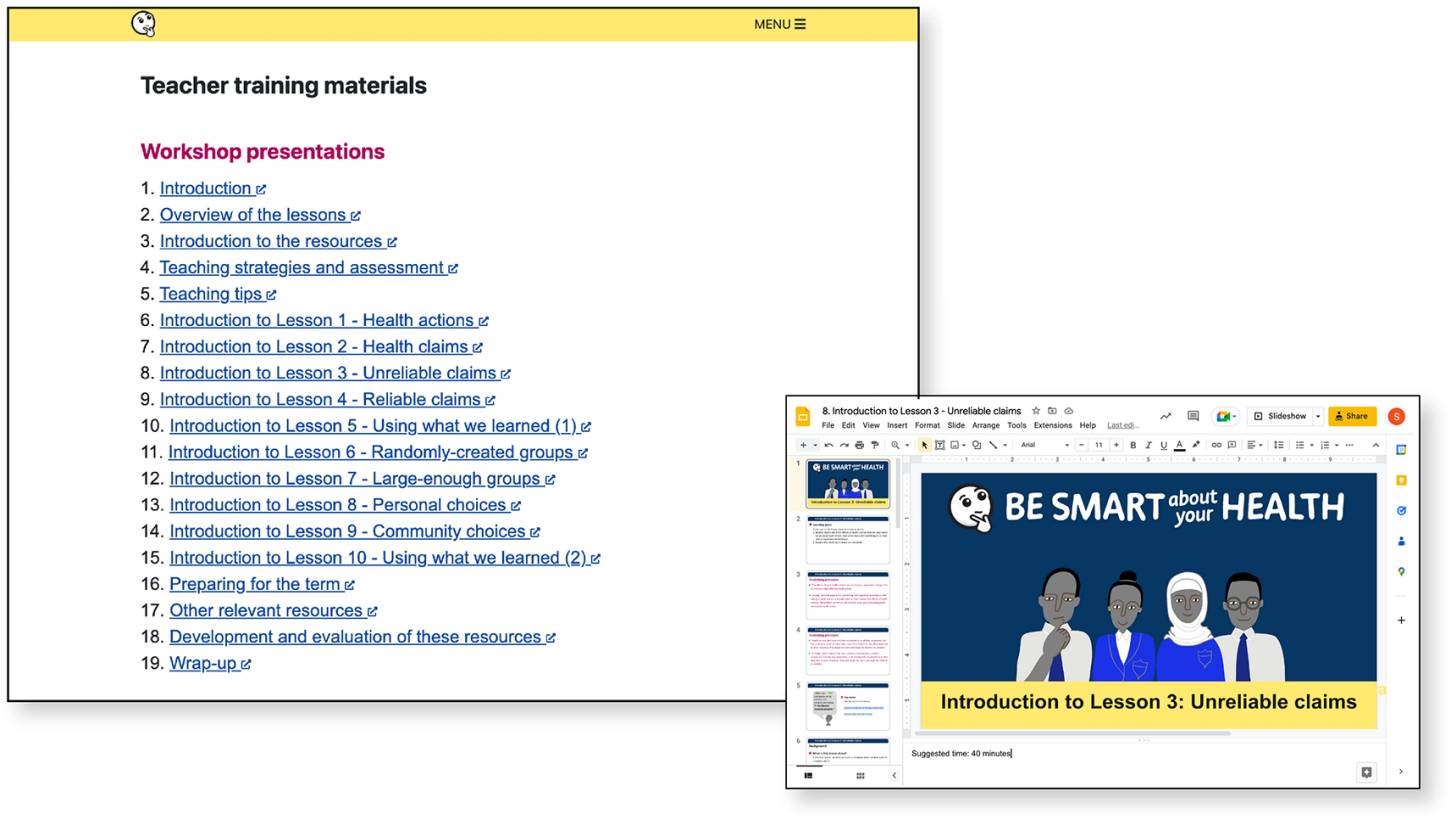
Teacher training materials.

**Figure 9.**
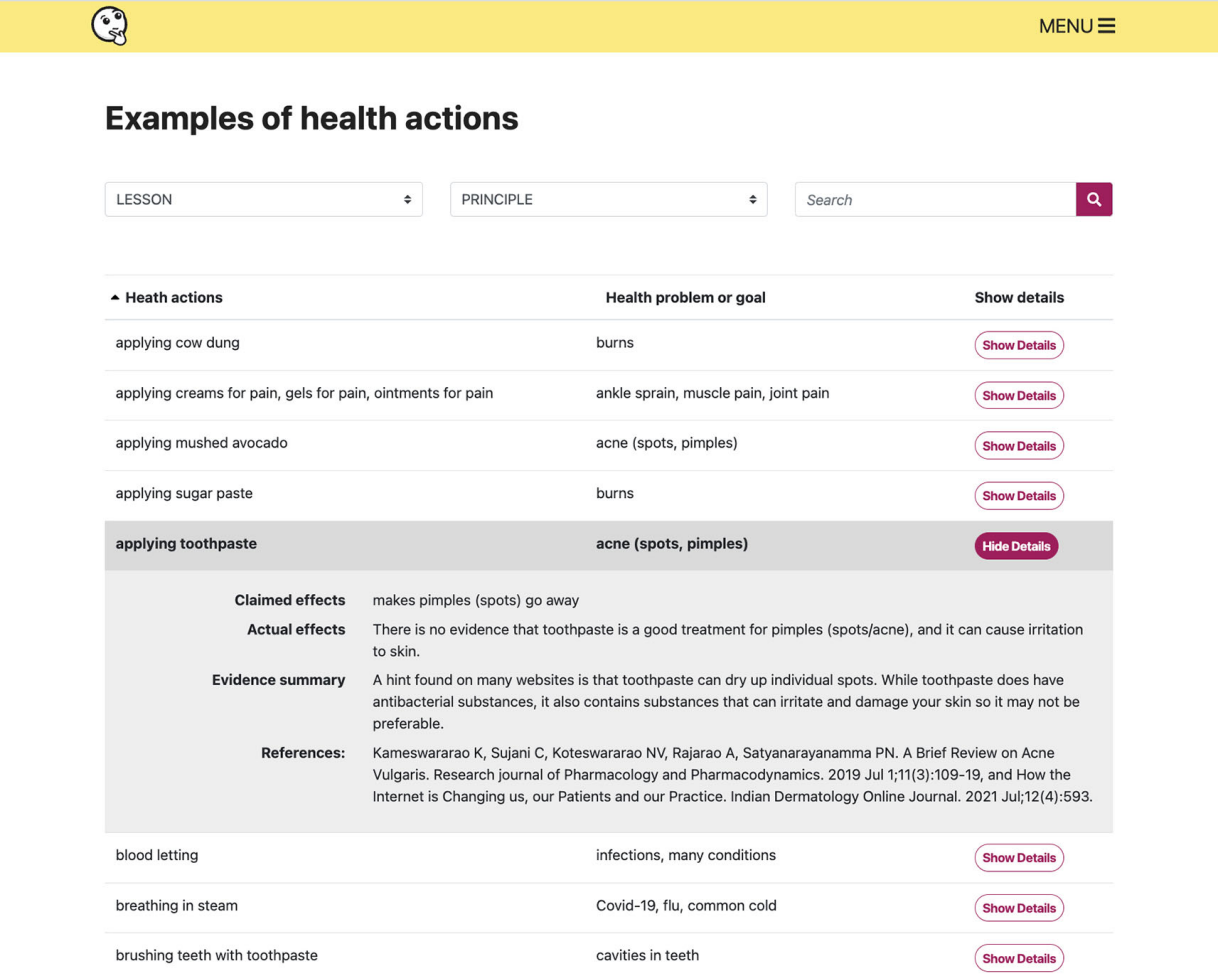
The examples collection provides alternative health actions or health problems/goals for each lesson or Key Concept in the resources.

The final set of resources, Be smart about your health, is an open access website for teachers comprised of ten lesson plans, a teachers’ guide, and extra resources including materials for teacher training. ^53^ Lesson plans are designed in two modes: for use in classrooms with a blackboard/flipchart (optimized for smartphone) and for classrooms with a projector with lessons formatted as downloadable Google Slides presentations. See Table 7 and Figures 4– 9.

**Table 7.** Final list of ten 40-minute lesson plans.

Lesson title	Description
**Lesson 1: Health actions**	Health actions, and why it is important to think carefully about them
**Lesson 2: Health claims**	Recognising claims about the effects of health actions
**Lesson 3: Unreliable claims**	Identifying some common types of unreliable claims
**Lesson 4: Reliable claims**	Identifying more reliable claims by considering whether the claim is based on a comparison of health actions
**Lesson 5: Using what we learned**	A review of the first four lessons, applying what has been learned to daily life, and recognising its limits
**Lesson 6: Randomly-created groups**	Assessing the reliability of comparisons by considering one important feature of reliable comparisons: randomly-created groups
**Lesson 7: Large-enough groups**	Continuing to assess the reliability of comparisons by considering whether the groups are large enough
**Lesson 8: Personal choices**	Thinking critically to make smart personal choices about health actions
**Lesson 9: Community choices**	Thinking critically to make smart community health choices
**Lesson 10: Using what we learned**	A review of all nine lessons, applying what has been learned to daily life, and recognising its limits

***Lessons plans:***

- *Content:* Set of ten lesson plans, two of which are for review and applying what students learned to their daily lives ( Table 7)
- *Structure:* Each Lesson Plan has an Overview, Lesson, and Background section. Each Lesson is designed to be taught in 40 minutes, and has three parts: Introduction, Activity, and Wrap-up ( Figure 5)
- *Format:* To accommodate for varied ICT infrastructure in schools, we created two modes of resources for teachers to deliver lessons: Blackboard lesson plans and Projector lesson plans. Blackboard lesson plans are optimised for teachers on mobile devices and work also offline. These can also serve as a back-up in the event of electricity outages. Projector mode is for use in classrooms that have access to a projector. We also created sets of optional, downloadable printouts for teachers to use as a paper back-up ( Figure 6)

***Teachers’ guide***: This includes an introduction to why the learning goals are important, a description of the content and how it ties to the curriculum in Kenya, Rwanda and Uganda, how to navigate the resources, how they were developed, and where to find other relevant resources ( Figure 7).

***Extra resources***:

- Teacher training materials, designed to be taught in workshops by teachers who participated in pilots ( Figure 8)
- Glossary
- Examples collection (alternative examples of health actions for each lesson) ( Figure 9)
- Teaching strategies for teaching critical thinking
- Underlying principles

### Findings from Phase 1: Groundwork

Below we describe output from the preliminary studies in Phase 1. As explained earlier, data from these studies have already been reported and discussed in other publications. However, for the reader to understand the full development process, and how these results influenced our objectives, content, and design, they are summarised below.

**Analysing contexts**

We organised key findings from the context analyses in four themes: *motivation* to teach or learn the content; *capability* to teach the content; *capability* to use digital resources; and *opportunity* to teach the content within existing curricula using digital resources. ^39^^–^^42^ Based on these findings, we identified opportunities and challenges, and made decisions about how to resolve those.

Stakeholders considered it to be important for students to learn to think critically about health, and the curricula included related learning goals. But teachers lacked capabilities and opportunities to teach both critical thinking in general, and about health choices more specifically. Additionally, schools’ digital infrastructures ranged from fully equipped with student computer labs to schools with few devices and no internet. Some of the strategies we decided to use included creating a teacher’s guide with in-depth background content and providing extra information about teaching strategies for critical thinking. We created lessons in different modes, with offline functionality to accommodate for variation in access to computers or digital infrastructure. See Table 8 for more detail about these findings and our solutions.

**Table 8.** Synthesis from context analyses and solutions.

Theme	Opportunities and challenges	Solutions
**Motivation to teach or learn the content**	Curricula in Kenya, Rwanda and Uganda included learning goals related to ‘critical thinking’ as a generic competence and to ‘health’, but none related specifically to ‘critical thinking about health’. Curriculum developers, teachers, and students said it was important for students to learn to think critically about health information and choices. However, teaching was largely exam-focused, so teachers and students were unlikely to prioritise content not included in exams.	In the teachers’ guide, we included descriptions of how the content mapped onto each country’s national curriculum. We communicated regularly with curriculum development offices to facilitate alignment with curricula, ownership of the resources, and future uptake of them.
**Capability to teach the content**	Teachers lacked prior knowledge of the IHC Key Concepts.	We created a teachers’ guide with an introduction and more detail about the IHC Key Concepts. In each lesson plan, we created a detailed background section describing the respective IHC Key Concepts for that lesson.
	Teacher said they lacked experience teaching and evaluating critical thinking in general. We did not identify any existing resources in use for learning or teaching critical thinking.	We created lessons drawing on teaching strategies for critical thinking that we identified in our overview of systematic reviews and included descriptions of these in lesson plans and Extra resources.
**Capability to use digital resources**	Teachers had varied experience using ICT for teaching. Many lacked ICT training.	To facilitate ease of access we developed open-access web-based resources. The design is responsive, therefore suitable for any screen size.
		To increase ease of use, we dropped log-in functionality and simplified the interface as much as possible. We used large font sizes, consistent formatting, and minimized amount of texts to facilitate ease of use during teaching.
		In the teachers’ guide, we included a help section explaining the navigation and technical features of the site.
		We created sets of optional, downloadable printouts for each lesson for teachers who preferred or had the opportunity of making paper copies.
**Opportunity to teach the content within existing curricula using digital resources**	We identified subjects in the Kenyan, Rwandan, and Ugandan curricula where the IHC Key Concepts could fit in the event of future uptake. Curriculum development offices would need to approve the use of any new teaching resource. Resources must be possible to download, adapt and republish on national platforms.	To facilitate tailored implementation, we created an adaptable, translatable solution using Google drive as an editing platform. Curriculum development offices can install their own versions of the resources for future translations or adaptations.
	Schools had very different levels of access to ICT infrastructure for teaching and learning, ranging from almost no access to well-equipped computer labs. About half of Rwandan secondary schools have “smart classrooms”: computer labs with laptops and Internet access. Poor Internet connectivity and unstable electricity were persistent barriers to use of digital resources in many schools, in all three countries.	To accommodate for varied access to ICT across schools, we created resources for delivering lessons in three different modes: Blackboard, Projector, and Student-computer modes. Blackboard lesson plans were optimised for teachers on mobile devices. These could also serve as a back-up in the event of electricity outages. Projector lesson plans included downloadable lessons formatted as Google Slides presentations. We also created sets of optional, downloadable printouts. Student-computer mode were designed for computer lab classrooms, with interactive lessons for students to use individually or in groups, in a class led by a teacher. (We later deactivated this mode, see results from development cycles below.)
		To accommodate users with a poor Internet connection, we built in offline functionality: when the user visits a page online, an automatic download begins, with messages and icons alerting the user. We also created a cached version that loads faster, for slow Internet connections.

**Prioritising IHC Key Concepts**

Twelve curriculum specialists, teachers, and researchers, in Kenya, Rwanda, and Uganda prioritised nine of the IHC Key Concepts ( Box 1) as suitable, relevant, and important for lower-secondary school students. ^54^ These nine concepts formed the basis for the learning goals and subsequent content development.

Box 1. **Nine prioritised IHC Key Concepts.**

• Treatments can cause harms as well as benefits.
• Widely used treatments or those that have been used for a long time are not necessarily beneficial or safe.
• Treatments that are new or technologically impressive may not be better than available alternatives.
• Personal experiences or anecdotes alone are an unreliable basis for most claims.
• Identifying effects of treatments depends on making comparisons.
• Comparison groups should be as similar as possible.
• Small studies may be misleading.
• Large, dramatic effects are rare.
• Weigh the benefits and savings against the harms and costs of acting or not.

**Identifying teaching strategies**

In an overview of systematic reviews, we identified teaching strategies for helping primary and secondary school students learn to think critically. ^44^ We experimented with using different strategies in each lesson, but found it added too much to teachers’ and students’ procedural cognitive load. Therefore, we chose a limited set to minimize variation and used most strategies across all lessons ( Box 2). Additionally, we prepared summaries of all the teaching strategies we considered using.

Box 2. **Teaching strategies included in lessons.**

Strategies used across all lessons:

‐ Guided notetaking
‐ Small group work
‐ Response cards
‐ Homework – collecting claims and choices about health actions
‐ Standard lesson structure
‐ Setting objectives and providing feedback
‐ Multimedia design

Strategies used in individual lessons:

‐ Concept mapping
‐ Concept cartoons
‐ Inquiry-based instruction
‐ Quiz
‐ Role play

For more detail, see also descriptions of teaching strategies we considered including (located under the Extra resources menu of the Besmarthealth.org website).

**Collecting examples**

To illustrate concepts, and make lessons more engaging, we used a broad range of examples in the lessons involving conditions and health actions that students said they found interesting. ^45^ We also created a searchable collection of relevant health actions and health problems or goals that can be used to illustrate key concepts, with plain language summaries of the evidence for each example ( Figure 9). The examples collection is part of the “Extra resources”.

### Findings from Phase 2: Development cycles

Using our findings from the Groundwork phase as a starting point, we conducted three development cycles of idea generation, prototyping, data collection and analysis, and stakeholder checking, which form the basis of this paper. The feedback was generally consistent regarding what people experienced as positive or problematic. Although there were far more positive findings, our main focus in collecting and reporting feedback was to identify problems that would require changes for the next iteration of the resources. We present the main findings thematically below.

***Positive, constructive findings***

Many teachers and students appreciated the learning in these lessons and experienced it as relevant both for other subjects and for their daily lives. Many students participated actively in class, including students who normally were less active. Many teachers felt the Projector mode of lessons worked best, providing structure and visual support to the lesson and helping to focus class attention. The blackboard mode and print-outs work satisfactorily for classes without projectors, or as a back-up in the event of power failure. Table 9 provides a detailed description of positive or constructive findings.

**Table 9.** Positive, constructive findings.

Theme	Description	Illustrative quotes or design implications
**Relevance to daily life**	Many teachers and students said the lessons were relevant to their daily lives and enjoyable.	*"I liked the idea that most of the lesson examples were daily life, we could easily relate."* (Student, group interview, Uganda) *“The lessons enabled the students to think of a personal choice and the advantages and disadvantages of making a certain decision”* (Teacher, Kenya Group Interview).
**Engaging and confidence-building for students**	Most students found the lessons engaging and that they increased their confidence. In the pilot lessons, students participated actively in quizzes and activities, including some who normally were quiet.	*“I am shy, but group work made me stand to present and this will give me confidence.”* (Student, group interview, Uganda)
**Structure and design of the resources**	For the most part, the structure of the resources and the lessons was appreciated.	*“I am impressed of how you present the content in a clear and easy-to-follow manner. I also like how you organize the concepts in main groups and subgroups, it gives an immediate understanding of the focus areas.”* (International advisory group member)
	In Rwanda, stakeholders favored a lesson structure that encourages “discovery”, where the teacher does not provide explanations before an activity. However, in Kenya, stakeholders favored a lesson structure where the teacher provides definitions and explanations were upfront, which are then reinforced through learning activities.	Design implication: We added a “discovery” question at the start of each lesson and provided definitions and explanations before the activity.
**Usefulness for thinking critically about health actions**	Many teachers and students said the resources were useful for thinking critically about health actions and in general.	*“It was nice and educative. It will help us not to just follow band wagon. Like we buy certain toothpaste but just because it is many friends use it.”* (Student, group interview, Uganda)
**Satisfaction with projector and blackboard modes**	The projector mode worked best, with the illustrated slides helping focus class attention, and aiding understanding. The blackboard mode also seemed to work satisfactorily.	*“Projector based lesson plans helped both teacher and students to focus on one activity than the computer-based version. There wasn't any interruption during the lesson.”* (Observation pilot lesson, Rwanda)
**Useful lesson printouts**	Some teachers found the printouts a useful way of continuing lessons even when there was a power outage preventing them from using a projector.	*“After some few minutes, the Power went off and the projector stopped showing the lesson. The teacher immediately gives the printouts to students to follow the lesson. After 3 minutes, the power returned.”* (Observation pilot lesson, Rwanda)

***Negative findings and how we addressed them***

We resolved problems as they appeared and changed the design of the prototypes dramatically from the first to the final development cycle. However, four main thematic challenges persisted throughout development: issues regarding the student-computer mode of lesson plans, Time, Comprehension, and Examples.

• Computers in classrooms took time to set up; equipment problems were common; students were often distracted by other online content; teachers found class discussions difficult to organise when students were using computers. Since teachers who had tried both the student-computer and Projector modes preferred the latter, we agreed with curriculum developers to drop the student-computer mode.
• School schedules lacked time to teach lessons that were not in the curriculum, and teachers struggled to complete lessons within the allotted 40 minutes. We simplified and shortened lesson content as much as possible, and developed modules for teacher training workshops to increase teachers’ confidence and capability to teach the lessons.
• Students had varying degrees of difficulty understanding lesson content, sometimes displaying a misunderstanding that was exactly the opposite of what they were intended to learn. We added “Common misunderstandings” to the lesson background, and designed lessons to include more review opportunities and informal assessment questions. We are exploring experiences and views of potential adverse effects in separate process evaluations ^30^^,^^31^^,^^55^ and in a qualitative evidence synthesis. ^56^
• We struggled finding appropriate examples of reliable and unreliable claims, as students often thought the lesson was about the example rather than the underlying Key Concept. Our solutions included finding a balance between real and fictional, well-labeled examples, guidance and prompts for teachers (including suggestions to find their own examples), and developing an example collection for teachers to find alternatives.

Tables 10- 13 provide a more detailed description of challenges and solutions.

**Table 10.** Difficulties using the Student-computer mode.

Description	Illustrative quotes	Design and content implications for the final version
Some curriculum developers wanted more media-rich, interactive resources that students could use for self-study in computer labs. However, we found technical, logistical, and behavioural problems with the student-computer mode: • Valuable teacher time used for setting up and charging the computers beforehand. • Broken computers. • Necessity for students to sometimes share a computer. • Students needing help finding browsers and reopening the resources after power outages. • Students cheating on quizzes or avoiding discussing things by opening the teacher resources or jumping ahead in the lessons to find the correct answers. • Students visiting other websites during the class. • Group discussions, one of the teaching strategies we had prioritized, not working well. Teachers who had tried both the student-computer and projector modes preferred the latter.	*“It is not easy for a teacher to handle technical issues while teaching. It disturbs her and the students also lose the focus. Most technical issues they experience, is to reopen the resources, to restart the computer, to help the students find the pages of where the teacher is focusing on, and the power issues.”* (Observation pilot lesson, Rwanda) *“The students were told to discuss in groups the activity part 1,2 and 3 but the students did not focus on doing these activities, they were busy exploring how the laptops work, visiting other websites, and others were writing the notes instead of discussing in groups.”* (Observation pilot lesson, Rwanda)	After the first few pilot lessons, in consultation with the curriculum developers, we decided to drop the student-computer mode.

**Table 11.** Time limitations.

Description	Illustrative quotes	Design and content implications for the final version
With early prototypes, lessons often took more than the intended 40 minutes because: • The content, including key terms, was often unfamiliar to students and teachers. • Some of the teaching strategies, such as getting students to take structured notes, and organising small group discussions, were time-consuming. In pilots, teachers tried to save time by skipping parts of, or entire, lessons or by falling back on didactic teaching strategies. Participating schools squeezed pilot lessons into their schedules at a time when schedules were already under extra pressure due to pandemic-related school closures. Classes were often held before or after regular school hours, when both teachers and students were tired.	*"I think the teacher should give us more time to think because you could just start thinking about an answer and the teacher says the 3 minutes are done"* (Student, group interview, Uganda)	We suggested reducing the number of IHC Key Concepts included. Due to resistance to this in stakeholder checking, we rearranged content and combined some lessons. We made a number of other timesaving changes: • removed quizzes and shortened reviews in most lessons, • limited activities to one per lesson, • used buzz-groups (2-3 students who are sitting next to each other) or whole-class discussions instead of small-group work, • developed printable student handouts and classroom posters to reduce students’ need to take notes.

**Table 12.** Problems with understanding.

Description	Illustrative quotes	Design and content implications for the final version
Some students did not understand some of the content. When asked to describe take-home messages from a lesson, students sometimes displayed a misunderstanding that was exactly the opposite of what they were intended to learn. Many students performed poorly on the test at the end of the pilot. Low scores in all three countries were likely in part linked to teachers’ lack of familiarity with the content lessons—especially concepts about research evidence. Results can be found in the dataset:“Pilot data and test results - analysis of problems and proposed solutions.pdf”. Teachers emphasized the need for checking students’ comprehension from lesson to lesson.	*“Whenever I go to buy a medicine, I need to check how long it has been in use and how many have used it”* (Rwanda, student interview) *“Personal experience can help to have information about any disease if you have experienced it”* (Rwanda, student interview)	To support students’ and teachers’ understanding: • We iteratively simplified the lesson structure, language, and content. • We added “Common misunderstandings” to each lesson Background. • We added review questions and short key messages at the end of lessons, which are repeated at the start of the next lesson. We developed printouts with definitions of key terms. To support teachers’ evaluation of students understanding and progress, we incorporated two review lessons (lessons 5 and 10) with quizzes. To support teachers’ understanding of the content and confidence in using the resources, we added a teacher training component to the intervention: a two-day workshop with presentations and activity materials, introducing teachers to the resources, teaching strategies, and content of each lesson, as well as providing teaching and preparation tips. We designed these training materials to be used by teachers who were already familiar with the resources (e.g., those who participated in pilots), so training would not be dependent on our team’s participation and would be scalable.

**Table 13.** Unsuitable or distracting examples illustrating the Key Concepts.

Description	Illustrative quotes	Design and content implications for the final version
Familiar examples engaged students, but often led to class discussions focused on the specific example rather than the underlying, generic concept that it illustrated. Some students thought that the lesson was about the information in the example, and not about learning to think critically about health information. On the other hand, fictional or unfamiliar examples appeared less engaging, because students could not relate them to their own lives or found them confusing. Many students did not readily question the reliability of claims about Covid-19 measures that were already widely implemented. Some teachers proposed that we add examples that were more familiar to students. In many cases, teachers supplemented with their own examples.	*“They take examples as key messages of the lesson they learnt.”* (Rwanda, teacher group interview) *“Student were not able to give examples of other possible options that could be made if someone doesn’t choose to cool a burn with running water. This example was not familiar to them.”* (Rwanda, observation pilot lesson) *“Some examples of Covid-19 used in these lessons limit students to think big. For example, if you are asking the students if wearing a mask is effective in reducing the spread of Covid-19, they will all say yes, because they are all using them, without even thinking if they are good to use or not.”* (Rwanda, teacher group interview) *“She just suggested adding more information about adolescent health and more examples of claims that are often told to the youth such as believing that sexual intercourse wipes away the pimples and lessens the menstrual cramps during the period.”* (Rwanda, teacher interview) *“The teacher used other examples that students seemed to relate with: use of marijuana and its effect on school dropout, anti-retroviral drugs and death rates, circumcision and HIV, and a community with drunkenness and community development.”* (Kenya, observation pilot lesson)	As far as possible, we used real, familiar, and relevant examples, drawing on examples collected from students. Where we were unable to identify a real example that seemed appropriate, we used fictional examples and tried to clearly indicate that they were fictional. In the teachers’ guide, we suggested teachers could use their own examples or find new ones in the searchable collection of health actions in the extra resources. In the teachers’ guide and the teacher training materials, we emphasized that the examples must illustrate the generic concepts, not just draw attention to specific health conditions, actions, or claims. In the background section of each lesson plan, we summarised research evidence about the used in that lesson. We also provided summaries of the evidence for the effects of all the health actions used in the resources in the searchable collection of health actions. We did this to ensure that the teachers were familiar with the examples, felt comfortable using them, and were prepared to prevent misunderstandings about the reliability of claims or evidence relating to them.

### Covid-19 consequences

The COVID-19 pandemic disrupted our work in many ways. At times, social-distancing and travel restrictions, as well as school closures, prohibited in-person meetings and data collection and piloting. When schools reopened, teachers were pressed to make up for lost time, sometimes rushing to finish a pilot lesson or not completing it. On the other hand, the abundance of unreliable claims about Covid-19 provided timely examples and motivation.

*“The lesson itself was very easy to understand because the students have already experienced what was used as example in the content (closing schools to reduce the spread of covid-19 infections).”* (Rwanda, Observation pilot lesson)

Workarounds to challenges included communicating with stakeholders individually via audio or video call, or chat, and conducting some of interviews with home networks.

## Discussion

Using a human-centred design approach, we developed adaptable, digital resources for teaching critical thinking about health in secondary schools in Kenya, Rwanda, and Uganda. In Phase 1 of the design, we gained insight into user and stakeholder needs, and the wider educational contexts. In Phase 2, we iteratively developed prototypes by generating ideas, prototyping, collecting and analysing data, and checking our ideas and analysis with stakeholders.

### Implications

In many countries, curriculum developers are moving away from traditional knowledge-based curricula to competency-based curricula, with critical thinking among the core competencies, ^57^ including in Kenya, Rwanda, and Uganda. ^39^^–^^41^ However, our context analyses showed that teachers lacked training and resources to teach critical thinking. ^39^^–^^41^

In their systematic review of strategies for teaching students to think critically, Abrami *et al.* found that three strategies were most effective, especially when combined: “the opportunity for dialogue, the exposure of students to authentic or situated problems and examples, and mentoring”. ^58^ Based on those findings, and findings from our overview of systematic reviews, ^44^ we focused on using authentic examples and discussion activities in our secondary school resources.

A review of education technology in low-income countries distinguishes between two categories of digital interventions: computer aided learning (CAL) and computer aided instruction (CAI). ^59^ Broadly speaking, CAL interventions are designed for the student-as-user, and either complement or supplement classroom instruction. CAI interventions, on the other hand, are designed for the teacher-as-user, and aim to enhance the quality of instruction. We developed and piloted resources for both CAL (the student-computer mode of lessons) and CAI (the blackboard and projector modes of lessons).

We found a variety of problems with the student-computer (CAL) resources. Many of the problems were logistical or technical, and not specific to our project, such as time needed to set up and charge computers, faulty or missing equipment, and unstable electricity. In addition, some curriculum developers (where student-computer resources were piloted) said that the resources lacked media-richness (such as animations or videos) as well as interactivity that we could not feasibly develop in this project and that teachers and students were possibly not experienced enough to use.

Regardless of the level of ICT preparedness, it might be that self-led study strategies and CAL interventions are not the best starting point for developing critical thinking skills, since it is more challenging to creating opportunities for dialogue when students are alone behind a screen, working at different paces from each other. In our case, developing functionality for dialogue to take place on the computer was not an option, due to technical barriers in the settings, such as lack or unreliable internet access. Computer-based mentoring ^60^^–^^63^ would require much more sophisticated programming than we had resources for. Moreover, less literate and less computer-literate students would be at a disadvantage. In their review, Abrami and colleagues’ found that discussion was especially effective when the teacher posed questions and there were whole-class, teacher-led discussions, or teacher-led group discussions. ^58^

Rwandan teachers who piloted both the student-computer and projector resources preferred the latter, with the blackboard and printed resources as a backup for when electricity failed. Besides being technically less demanding, the slides seemed to better activate students, support small and large-group discussion, and focus collective attention. Moreover, teachers can download and tailor slides. The lower-tech CAI modes require much less investment in equipment than high-tech CAL modes and could dramatically lower the threshold for bringing digital learning resources that support critical thinking into low-resource classrooms.

### Strengths and limitations

Strengths of this study include comprehensive groundwork; several development cycles; including pilots of all lessons in three countries; input from a wide variety of stakeholders and participants; systematic methods used to prioritise content and identify effective teaching strategies; data collection using complementary, mixed methods; independent initial coding and analysis by more than one researcher; and stakeholder checking to triangulate analyses.

A limitation is that the project team developed the prototypes and collected and coded data. This might have biased participants to give positive feedback, or introduced bias in the analysis, leading to an exaggeration of positive feedback or lack of attention to negative feedback. We took measures to mitigate this: the interview guide included questions that explicitly encouraged participants to provide negative feedback; data was collected in three countries and coding in each country was reviewed by two researchers in order to reduce potential for bias; we introduced “stakeholder checking” to triangulate our data analyses and check that the solutions we developed were appropriate.

It is possible that the amount of data we collected placed an undue burden on participants and other stakeholders. We are exploring this in a separate stakeholder evaluation. ^38^ It is also possible that the prototypes caused harm to some students who misunderstood concepts or examples. We are exploring potential adverse effects of the final resources in process evaluations ^30^^,^^31^^,^^55^ and a qualitative evidence synthesis. ^56^

*Reflexivity considerations*

The core project team all have backgrounds as public health researchers, while the stakeholders recruited for the purposes of collaborating and providing feedback have mostly backgrounds in education with no previous engagement in this research topic. The core project team members are from many different settings, while all stakeholders are from East Africa with the exception of the international advisory group. The PhD fellows and several other core team members were working full-on this project, and therefore highly invested in a positive outcome, while stakeholders were participating voluntarily, in addition to their other full-time work or study commitments.

We have tried to be mindful of our differing agendas, cultural perspectives, and the ways in which the core team’s ambitions might influence the outcome of the work or place undue burden on the participants. We discussed these topics frequently during weekly core team meetings and have also explored some of these questions using more structured methods, designed as separate studies. The context analyses we conducted at the beginning of this work helped us view our research agenda from the perspective of teachers and curriculum developers and understand whether and how our research might better align with existing objectives within the three educational systems. The stakeholder engagement assessment (manuscript under development) provided stakeholders with opportunities to give us feedback about how they experienced their participation while it was ongoing. The qualitative evidence synthesis of post-trial process evaluations (manuscript under development) will help us understand if there have been any adverse effects of the resources, seen from the point of view of the participants.

Relationships between the core team and recruited stakeholders developed over time, and some people who were initially recruited as participants became engaged to the degree that they fulfilled requirements for co-authorship of this study.

## Conclusion

Using a human-centred design approach, we created adaptable, digital resources for teaching lower secondary school students to think critically about health actions in ten 40-minute lessons. Teachers and students in Kenya, Rwanda and Uganda found the lessons relevant and useful. The open access resources are free to use and can be translated and adapted to other settings. Designed to accommodate use in classrooms with differing digital infrastructure, such as lack of internet connectivity or unstable electricity, they include materials in two modes for classrooms equipped with blackboards or projectors. Teachers with access to a projector preferred the projector resources. In addition to the lessons, resources include a teachers’ guide, glossary, a searchable set of alternative examples of health actions, summaries of teaching strategies for critical thinking, and teacher training materials. The Covid-19 pandemic disrupted the design of the resources, but also provided timely examples and motivation to learn.

Next research steps are already in progress, including randomised trials to evaluate the effect of using the resources on students’ ability to think critically about health actions, parallel process evaluations and one-year follow up studies.

## Data Availability

Zenodo. Dataset for “Teaching critical thinking about health information and choices in secondary schools: human-centred design of digital resources”, https://doi.org/10.5281/zenodo.7695782. ^47^ This project contains the following underlying data:
•
DevelopmentCycle1__data feedback and observations.csv (Coded datapoints extracted in Development cycle 1 from individual/group interviews with teachers/students and observations of lesson pilots in Rwanda, Kenya and Uganda) •
DevelopmentCycle1_internal team feedback.csv (Feedback, comments, suggestions from our research team during Development cycle 1) •
DevelopmentCycle1_interntl-advisory-grp-dec-jan-2020.csv (First round of feedback from international advisory group, via email) •
DevelopmentCycle1_interntl-advisory-grp-jun-aug-2020.csv (Second round of feedback from international advisory group, via email) •
DevelopmentCycle1_interntl-advisory-grp-proto1.2.csv (Third round of feedback from international advisory group, via email) •
DevelopmentCycle1_legend.csv (Description of implications codes we used to assess the importance of observations and feedback for the next iteration of the resources, e.g., “Problem/Showstopper/A problem that we should address”) in Development Cycle 2 •
DevelopmentCycle2__data feedback and observations data.csv (Coded datapoints extracted in Development cycle 2 from individual/group interviews with teachers/students and observations of lesson pilots in Rwanda, Kenya and Uganda,) •
DevelopmentCycle2_codes and response options.csv (List of codes/response options used in Development cycle 2 to code or describe datapoints) •
DevelopmentCycle2_definitions.csv (Description of “nature”, “implications” and “Action for resolving” codes used in Development cycle 2) •
DevelopmentCycle2_internal team feedback.csv (Feedback, comments, suggestions from our research team during Development cycle 2) •
Pilot data and test results - analysis of problems and proposed solutions.pdf (Analysis of problems and proposed solutions after lesson pilots at the end of Development cycle 2 that we presented to curriculum developers for stakeholder checking and input) Zenodo. Extended dataset and Coreq Checklist for “Teaching critical thinking about health information and choices in secondary schools: human-centred design of digital resources”, https://doi.org/10.5281/zenodo.7806139. ^48^ This project contains the following extended data:
•
COREQ_checklist New.pdf (COREQ checklist for this article) •
Group Interview guide Post-Pilot - Students Group interview guide.docx (Guide used as a basis for interviewing groups of students after they had participated in piloting lessons at the end of Development cycle 2) •
Group Interview guide Post-Pilot - Teachers.docx (Guide used as a basis for interviewing teachers after they had piloted lessons at the end of Development cycle 2) •
Interveiw guide Early - Student network.docx (Guide used as a basis for interviewing students in Development cycle 1) •
Interview guide Early - Curriculum develop Questionnaire.docx (Guide used as a basis for interviewing curriculum developers in Development cycle 1) •
Interview guide Pilot - Teachers User testing interview guide.docx (Guide used as a basis for user test interviews with participating teachers in pilot lessons at the end of Development cycle 2) •
Interview Guide Pre-pilot - Teacher-Student - User test.docx (Guide used as a basis for user test interviews of students and teachers before they participated in pilot lessons at the end of Development Cycle 2) •
Observation form Pilot - Observers.docx (Form used by observers to structure observation data of pilot lessons) Data are available under the terms of the Creative Commons Attribution 4.0 International Public License.

## References

[1] Oxman M Larun L Gaxiola GP : Quality of information in news media reports about the effects of health interventions: systematic review and meta-analyses. *F1000Res.* 2021;10:10. 10.12688/f1000research.52894.1 PMC8756300 35083033

[2] Boutron I Haneef R Yavchitz A : Three randomized controlled trials evaluating the impact of “spin” in health news stories reporting studies of pharmacologic treatments on patients’/caregivers’ interpretation of treatment benefit. *BMC Med.* 2019;17(1):1–10. 31159786 10.1186/s12916-019-1330-9 PMC6547451

[3] Dahlgren A Furuseth-Olsen K Rose CJ : The Norwegian public’s ability to assess treatment claims: results of a cross-sectional study of critical health literacy. *F1000Res.* 2021;9(179):179. 10.12688/f1000research.21902.2 38585673 PMC10995534

[4] Li HO-Y Bailey A Huynh D : YouTube as a source of information on COVID-19: a pandemic of misinformation? *BMJ Glob. Health.* 2020;5(5):e002604. 10.1136/bmjgh-2020-002604 32409327 PMC7228483

[5] Sharp MK Forde Z McGeown C : Irish media coverage of COVID-19 evidence-based research reports from one national agency. *Int. J. Health Policy Manag.* 2021;11:2464–2475. 10.34172/ijhpm.2021.169 35042323 PMC9818095

[6] Wagner DN Marcon AR Caulfield T : “Immune Boosting” in the time of COVID: selling immunity on Instagram. *Allergy, Asthma Clin. Immunol.* 2020;16(1):1–5. 32905318 10.1186/s13223-020-00474-6 PMC7468087

[7] Janiaud P Axfors C Hooft J van't : The worldwide clinical trial research response to the COVID-19 pandemic - the first 100 days [version 2; peer review: 2 approved]. *F1000Res.* 2020;9(1193). 10.12688/f1000research.26707.1 PMC7539080 33082937

[8] Jones CW Woodford AL Platts-Mills TF : Characteristics of COVID-19 clinical trials registered with ClinicalTrials. gov: cross-sectional analysis. *BMJ Open.* 2020;10(9):e041276. 10.1136/bmjopen-2020-041276 PMC7500290 32948577

[9] Glasziou PP Sanders S Hoffmann T : Waste in covid-19 research. *BMJ.* 2020;369:m1847. 10.1136/bmj.m1847 32398241

[10] Pian W Chi J Ma F : The causes, impacts and countermeasures of COVID-19 “Infodemic”: A systematic review using narrative synthesis. *Inf. Process. Manag.* 2021;58(6):102713. 10.1016/j.ipm.2021.102713 34720340 PMC8545871

[11] Borges do Nascimento IJ Pizarro AB Almeida JM : Infodemics and health misinformation: a systematic review of reviews. *Bull. World Health Organ.* 2022;100(9):544–561. 10.2471/BLT.21.287654 36062247 PMC9421549

[12] Chou W-YS Gaysynsky A Vanderpool RC : The COVID-19 Misinfodemic: moving beyond fact-checking. *Health Educ. Behav.* 2021;48(1):9–13. 10.1177/1090198120980675 33322939 PMC8685465

[13] Cusack L Del Mar CB Chalmers I : Educational interventions to improve people’s understanding of key concepts in assessing the effects of health interventions: a systematic review. *Syst. Rev.* 2018;7(1):68. 10.1186/s13643-018-0719-4 29716639 PMC5930693

[14] Roozenbeek J Linden S van der Goldberg B : Psychological inoculation improves resilience against misinformation on social media. *Sci. Adv.* 2022;8(34):eabo6254. 10.1126/sciadv.abo6254 36001675 PMC9401631

[15] Nsangi A Semakula D Rosenbaum SE : Development of the informed health choices resources in four countries to teach primary school children to assess claims about treatment effects: a qualitative study employing a user-centred approach. *Pilot Feasibility Stud.* 2020;6(18):18. 10.1186/s40814-020-00565-6 32055405 PMC7008535

[16] Semakula D Nsangi A Oxman M : Development of mass media resources to improve the ability of parents of primary school children in Uganda to assess the trustworthiness of claims about the effects of treatments: a human-centred design approach. *Pilot Feasibility Stud.* 2019;5(1):1–17. 10.1186/s40814-019-0540-4 31890267 PMC6935490

[17] Nsangi A Semakula D Oxman AD : Effects of the Informed Health Choices primary school intervention on the ability of children in Uganda to assess the reliability of claims about treatment effects: a cluster-randomised controlled trial. *Lancet.* 2017;390(10092):374–388. 10.1016/S0140-6736(17)31226-6 28539194

[18] Semakula D Nsangi A Oxman AD : Effects of the Informed Health Choices podcast on the ability of parents of primary school children in Uganda to assess claims about treatment effects: a randomised controlled trial. *Lancet.* 2017;390(10092):389–398. 10.1016/S0140-6736(17)31225-4 28539196

[19] Nsangi A Semakula D Oxman AD : Effects of the Informed Health Choices primary school intervention on the ability of children in Uganda to assess the reliability of claims about treatment effects, 1-year follow-up: a cluster-randomised trial. *Trials.* 2020;21(1):1–22. 10.1186/s13063-019-3960-9 31907013 PMC6945419

[20] Semakula D Nsangi A Oxman AD : Effects of the Informed Health Choices podcast on the ability of parents of primary school children in Uganda to assess the trustworthiness of claims about treatment effects: one-year follow up of a randomised trial. *Trials.* 2020;21(1). 10.1186/s13063-020-4093-x PMC7023790 32059694

[21] Austvoll-Dahlgren A Oxman AD Chalmers I : Key concepts that people need to understand to assess claims about treatment effects. *J. Evid. Based Med.* 2015;8(3):112–125. 10.1111/jebm.12160 26107552

[22] Chalmers I Oxman AD Austvoll-Dahlgren A : Key concepts for informed health choices: a framework for helping people learn how to assess treatment claims and make informed choices. *BMJ Evid. Based Med.* 2018;23(1):29–33. 10.1136/ebmed-2017-110829 29367324

[23] Austvoll-Dahlgren A Semakula D Nsangi A : Measuring ability to assess claims about treatment effects: the development of the 'Claim Evaluation Tools'. *BMJ Open.* 2017;7(5):e013184. 10.1136/bmjopen-2016-013184 28515181 PMC5777467

[24] Informed Health Choices: Informed Health Choices.n.d. Reference Source

[25] Nsangi A Semakula D Glenton C : Informed health choices intervention to teach primary school children in low-income countries to assess claims about treatment effects: process evaluation. *BMJ Open.* 2019;9(9):e030787. 10.1136/bmjopen-2019-030787 31511291 PMC6747654

[26] Semakula D Nsangi A Oxman A : Informed Health Choices media intervention for improving people’s ability to critically appraise the trustworthiness of claims about treatment effects: a mixed-methods process evaluation of a randomised trial in Uganda. *BMJ Open.* 2019;9(12):e031510. 10.1136/bmjopen-2019-031510 31852697 PMC6937069

[27] Effects of the Informed Health Choices secondary school intervention on the ability of lower secondary students in Kenya to think critically about health information and choices: Protocol for a cluster-randomized trial. *Zenodo.* 2022 [cited November, 2022].

[28] Effects of using the Informed Health Choices digital secondary school resources on the ability of Rwandan students to think critically about health: protocol for a cluster-randomised trial. *Zenodo.* 2022 [cited November, 2022].

[29] Does the use of the Informed Health Choices teaching resources improve the secondary students' ability to critically think about health in Uganda? A cluster randomised trial protocol. 2022 [cited November 2022].

[30] Ssenyonga R Lewin S Nakyejwe E : Informed heath choices intervention to teach secondary school adolescents in Uganda to assess claims about treatment effects: a process evaluation protocol. 2022. 10.5281/zenodo.6984730

[31] Mugisha M Nyirazinyoye L Oxman AD : Use of the Informed Health Choices digital resources for teaching lower secondary school students in Rwanda to think critically about health: protocol for a process evaluation. 2022.

[32] Chesire F Kaseje M Ochieng M : Effect of the Informed Health Choices digital secondary school resources on the ability of lower secondary students in Kenya to critically appraise health claims: protocol for a process evaluation. 2022 Feb 27, 2023. Reference Source

[33] Rosenbaum S Oxman M Oxman AD : Human-centred design development of Informed Health Choices (IHC) learning resources for secondary school students: Protocol. *Zenodo.* 2019 2019/12. Reference Source

[34] Rosenbaum SE : *Improving the user experience of evidence: A design approach to evidence-informed health care.* Oslo: Arkitektur- og designhøgskolen i Oslo;2010.

[35] Giacomin J : What is Human Centred Design? *Des. J.* 2014;17(4,2014):606–623. 10.2752/175630614X14056185480186

[36] Melles M Albayrak A Goossens R : Innovating health care: key characteristics of human-centered design. *Int. J. Qual. Health Care.* 2021;33(Supplement_1):37–44. 10.1093/intqhc/mzaa127 33068104 PMC7802070

[37] ISO: *Ergonomics of human-system interaction — Part 210: Human-centred design for interactive systems.* Geneva:2019.

[38] Nsangi A Oxman AD Oxman M : Protocol for assessing stakeholder engagement in the development and evaluation of the Informed Health Choices resources teaching secondary school students to think critically about health claims and choices. *PLoS One.* 2020;15(10):e0239985. 10.1371/journal.pone.0239985 33045009 PMC7549807

[39] Chesire F Ochieng M Mugisha M : Contextualizing critical thinking about health using digital technology in secondary schools in Kenya: a qualitative analysis. *Pilot Feasibility Stud.* 2022;8(1):227. 10.1186/s40814-022-01183-0 36203201 PMC9535840

[40] Mugisha M Uwitonze AM Chesire F : Teaching critical thinking about health using digital technology in lower secondary schools in Rwanda: A qualitative context analysis. *PLoS One.* 2021;16(3):e0248773. 10.1371/journal.pone.0248773 33750971 PMC7984628

[41] Ssenyonga R Sewankambo NK Mugagga SK : Learning to think critically about health using digital technology in Ugandan lower secondary schools: A contextual analysis. *PLoS One.* 2022;17(2):e0260367. 10.1371/journal.pone.0260367 35108268 PMC8809610

[42] Michie S Van Stralen MM West R : The behaviour change wheel: a new method for characterising and designing behaviour change interventions. *Implement. Sci.* 2011;6(1):1–12. 21513547 10.1186/1748-5908-6-42 PMC3096582

[43] Agaba JJ Chesire F Mugisha M : Prioritisation of Informed Health Choices (IHC) key concepts to be included in lower secondary school resources: A consensus study. *PLoS One.* 2023;18(4):e0267422. 10.1371/journal.pone.0267422 37027357 PMC10081733

[44] Oxman AD Dahlgren A García LM : *The effects of teaching strategies on learning to think critically in primary and secondary schools: protocol for an overview of systematic reviews.* Norwegian Institute of Public Health;2019.

[45] Moberg J : Creating an examples collection for the “Be Smart About Your Health” resources: part of the CHOICE project. 2023. 10.5281/zenodo.7702264

[46] Birt L Scott S Cavers D : Member checking: a tool to enhance trustworthiness or merely a nod to validation? *Qual. Health Res.* 2016;26(13):1802–1811. 10.1177/1049732316654870 27340178

[47] Rosenbaum S : Dataset for “Teaching critical thinking about health information and choices in secondary schools: human-centred design of digital resources”.[Data set]. *Zenodo.* 2023. 10.5281/zenodo.7695782 PMC11377934 39246586

[48] Rosenbaum S : Extended dataset and Coreq Checklist for "Teaching critical thinking about health information and choices in secondary schools: human-centred design of digital resources". *Zenodo.* 2023. 10.5281/zenodo.7806139 PMC11377934 39246586

[49] Charters E : The use of think-aloud methods in qualitative research an introduction to think-aloud methods. *Brock Educ. J.* 2003;12(2). 10.26522/brocked.v12i2.38

[50] Ericsson K Simon HA : Verbal reports as data. *Psychol. Rev.* 1980;87(3):215–251. 10.1037/0033-295X.87.3.215

[51] Kuniavsky M : *Observing the User Experience: A Practitioner's Guide to User Research.* 1 ed. Kaufmann M , editor. Morgan Kaufmann;2003;575p.

[52] Dahlgren A Semakula D Chesire F : Critical thinking about treatment effects in Eastern Africa: development and evaluation of an assessment tool using Rasch analysis. *F1000Res.* 2022 (In press).

[53] Informed Health Choices project: Be Smart About Your Health 2022 [Educational resources]. Reference Source

[54] Agaba JJ Chesire F Michael M : Prioritisation of Informed Health Choices (IHC) Key Concepts to be included in lower-secondary school resources: a consensus study. 2022. 10.1371/journal.pone.0267422 PMC10081733 37027357

[55] Chesire F Kaseje M Ochieng M : Effect of the Informed Health Choices digital secondary school resources on the ability of lower secondary students in Kenya to critically appraise health claims: protocol for a process evaluation. 2022.

[56] Oxman M Oxman AD Fretheim A : Participants' and investigators' experiences and views of potential adverse effects of an educational intervention: Protocol for a qualitative evidence synthesis (Version 1). 2023. 10.5281/zenodo.7681365

[57] Voogt J Roblin NP : A comparative analysis of international frameworks for 21st century competences: Implications for national curriculum policies. *J. Curric. Stud.* 2012;44(3):299–321. 10.1080/00220272.2012.668938

[58] Abrami PC Bernard RM Borokhovski E : Strategies for teaching students to think critically: A meta-analysis. *Rev. Educ. Res.* 2015;85(2):275–314. 10.3102/0034654314551063

[59] Rodriguez-Segura D : Educational technology in developing countries: A systematic review. University of Virginia EdPolicy Works Working Papers. 2020. Reference Source

[60] Gerard L Matuk C McElhaney K : Automated, adaptive guidance for K-12 education. *Educ. Res. Rev.* 2015;15:41–58. 10.1016/j.edurev.2015.04.001

[61] Xu Z Wijekumar K Ramirez G : The effectiveness of intelligent tutoring systems on K-12 students' reading comprehension: A meta-analysis. *Br. J. Educ. Technol.* 2019;50(6):3119–3137. 10.1111/bjet.12758

[62] Ma W Adesope OO Nesbit JC : Intelligent tutoring systems and learning outcomes: A meta-analysis. *J. Educ. Psychol.* 2014;106(4):901–918. 10.1037/a0037123

[63] Steenbergen-Hu S Cooper H : A meta-analysis of the effectiveness of intelligent tutoring systems on K–12 students’ mathematical learning. *J. Educ. Psychol.* 2013;105(4):970–987. 10.1037/a0032447

