# Supplementary material for: Teaching critical thinking about health information and choices in secondary schools: human-centred design of digital resources
Source: F1000Res. 2024 Sep 4;12:481. Originally published 2023 May 11. [Version 3] doi: 10.12688/f1000research.132580.3 (PMC11377934; doi:10.12688/f1000research.132580.3)
Supplement: Peer review history

# Reviewer report: Reviewer response for version 2

**DOI:** 10.5256/f1000research.163450.r250510

**Published:** 2024-05-14

**Authors:** Cynthia Baur

**Affiliations:** 1 University of Maryland at College Park, College Park, Maryland, USA

The authors provide a detailed description of a multi-phase and multi-step process to develop and test learning modules about health information and health choices for children aged 13-16 in Kenya, Rwanda, and Uganda. The project was developed and tested in partnership with many stakeholders in the three countries. The Introduction opens with a brief discussion of the many challenges of identifying accurate information but ends with a purpose statement that the article will describe the development of the learning modules. The rest of the paper is about the module development process with Methods and Results being about the modules and learning techniques. Although the paper includes a discussion of teaching strategies in the Results section, what is missing is a theory of learning and expected results that would help explain why the team chose the human-centered design approach that it did, along with some literature justifying this choice. The paper provides a lot of detail about how a human-centered design approach collects feedback and iterates prototypes, but I am unclear why a human-centered design approach was necessary to produce this set of modules. I am also unclear about the lessons the authors want to impart to readers. Should we always use a human-centered design approach for health curriculum for children of these ages? Under what conditions or purposes might a different approach be better? Instead of naming the products developed as the primary results, I expected to read about how well or not a human-centered approach had worked to produce these products. I also lost the thread of what happened to all the interview, group discussion and stakeholder data. The authors provide a rich description of their development process, but I'm not sure what they intend for readers to take away from this report of their process and products.

Is the work clearly and accurately presented and does it cite the current literature?

Partly

If applicable, is the statistical analysis and its interpretation appropriate?

Not applicable

Are all the source data underlying the results available to ensure full reproducibility?

Yes

Is the study design appropriate and is the work technically sound?

Yes

Are the conclusions drawn adequately supported by the results?

Partly

Are sufficient details of methods and analysis provided to allow replication by others?

Partly

Reviewer Expertise:

Health literacy, health communication, risk communication

I confirm that I have read this submission and believe that I have an appropriate level of expertise to confirm that it is of an acceptable scientific standard, however I have significant reservations, as outlined above.

# Response

**Published:** 2024-08-26

**Authors:** Sarah Rosenbaum

**Affiliations:** Norwegian Institute of Public Health, Norway

Peer review 2 and our corresponding responses:

**1) PEER REVIEW: **The Introduction opens with a brief discussion of the many challenges of identifying accurate information but ends with a purpose statement that the article will describe the development of the learning modules. Although the paper includes a discussion of teaching strategies in the Results section, what is missing is a theory of learning and expected results that would help explain why the team chose the human-centered design approach that it did, along with some literature justifying this choice. The paper provides a lot of detail about how a human-centered design approach collects feedback and iterates prototypes, but I am unclear why a human-centered design approach was necessary to produce this set of modules.

**RESPONSE: **I agree that addressing this would improve the manuscript, and have added three new sections to the Discussion: “Reflections about learning theory”, “Why a human-centred design approach”, and “Strengths and limitations of the human-centred design approach”, as well as a number of new references.

**2) PEER REVIEW: **I am also unclear about the lessons the authors want to impart to readers. The authors provide a rich description of their development process, but I'm not sure what they intend for readers to take away from this report of their process and products.

**RESPONSE: **See response #4 below about results.

**3) PEER REVIEW: **Should we always use a human-centered design approach for health curriculum for children of these ages? Under what conditions or purposes might a different approach be better?

**RESPONSE: **Educating young people in low-resource school settings to think critically about health choices is a complex problem. Developing instructional material for simpler and more well-defined learning tasks might be approached by less resource-intensive methods. I have added a text elaborating on this issue under Discussion: “Why a human-centred design approach”.

**4) PEER REVIEW: **Instead of naming the products developed as the primary results, I expected to read about how well or not a human-centered approach had worked to produce these products.

**RESPONSE: **“Results” are not only what we found when collecting and analysing data, but also the final solution we created and the noteworthy design decisions we made along the way. These three types of findings can be of interest to a range of readers: people who are designing educational resources based on IHC Key Concepts, people who are designing related educational resources, such as for teaching critical thinking or for use in low-resource learning environments, or people interested in the design approach more generally within the context of a research inquiry.

 However, I agree that we could have also included more explicit findings related to the human-centred design approach. Bazzano et al (2020) proposed a reporting guideline when reporting health research involving design that suggests reporting the following from design activities in the results section:

- What was designed
- Summary of major insights or reflections from the design process
- Description of decisions made during the design process

The guidance also suggests reflecting on the application of design to the work, including strengths and limitations. I have added a completed guidance checklist to the Extended data set, which indicates where in the manuscript each item can be found. Additionally, I am making the following changes to the manuscript:

 "Results" section changes:

- Made changes to the 1st paragraph in Results section to more clearly indicate the type of results we are reporting
- Added heading and text: “Other noteworthy design decisions”
- Made changes to some subheadings in the Results sections
- Made changes to some column headings in Tables 9-13, to clarify where results about "design decisions" could be found

"Discussion" section changes:

- Added “Strengths and limitations of the human-centred design approach for this work”

"Conclusion" section changes

- Adjusted text to differentiate more clearly between the three types of results

**5) PEER REVIEW: **I also lost the thread of what happened to all the interview, group discussion and stakeholder data.

**RESPONSE: **Our data is published on Zenodo. See list of data “Underlying data”, reference 47. However, I acknowledge that the documentation of a project of this nature can be difficult to follow. Therefore I’ve added content to the Discussion addressing this under the heading:

-  “Strengths and limitations of the human-centred design approach”

# Reviewer report: Reviewer response for version 1

**DOI:** 10.5256/f1000research.145511.r250515

**Published:** 2024-09-05

**Authors:** Catherine Mathews

**Affiliations:** 1 South African Medical Research Council, Cape Town, South Africa

This manuscript describes the processes undertaken to develop a set of digital learning resources to teach students in Kenyan, Rwandan and Ugandan schools to think critically about health and health care, and to develop skills to assess the reliability of claims about interventions to improve health. 

 Interventions to teach critical thinking are seldom included in the curricula of schools in the countries in which this research was conducted, despite that critical thinking is recognized as a valuable skill for young people to acquire, which will help them make decisions through the life course.  In the countries in which the research was conducted, and in many other countries, teachers lack skills and resources to enable them to teach critical thinking.

 Both the intervention and the measure selected to evaluate the effects of the intervention are based on a large body of sound research which is referenced in the manuscript.

 The manuscript provides valuable information about how to use a human-centered design approach to adapt a program that promotes critical thinking about health and to adapt measures to evaluate the program.  Stakeholders contributed to the intervention development process is described in sufficient detail and is appropriate.  The changes made to the interventions in response to stakeholders’ input are detailed. The problems identified during the design process, and the actions taken to address such problems indicate how feedback from stakeholders was used to inform the development cycles of the intervention. 

 The description of the set of lessons is clear and valuable and together with information about the key concepts that were taught in a sample lesson helps the reader gain more information about the intervention.

 The manuscript is written clearly, and the research processes are described comprehensively. It is rare to read a manuscript in which the intervention development process is set out systematically and clearly. The manuscript is excellent, and I do not have any suggestions for improvement.

**Major points**

 None

**Minor points**

 None

Is the work clearly and accurately presented and does it cite the current literature?

Yes

If applicable, is the statistical analysis and its interpretation appropriate?

Not applicable

Are all the source data underlying the results available to ensure full reproducibility?

Yes

Is the study design appropriate and is the work technically sound?

Yes

Are the conclusions drawn adequately supported by the results?

Yes

Are sufficient details of methods and analysis provided to allow replication by others?

Yes

Reviewer Expertise:

Sexual and reproductive health

I confirm that I have read this submission and believe that I have an appropriate level of expertise to confirm that it is of an acceptable scientific standard.

# Reviewer report: Reviewer response for version 1

**DOI:** 10.5256/f1000research.145511.r173757

**Published:** 2023-06-01

**Authors:** Anke Steckelberg

**Affiliations:** 1 Interdisciplinary Center for Health Sciences, Institute of Health and Nursing Science, Martin-Luther-Universitat Halle-Wittenberg, Halle (Saale), Saxony-Anhalt, Germany

The authors reported the development of digital resources to teach secondary school students applying a human-centred design. This project is part of a larger project comprising extensive exploration of stakeholders perspectives and contexts and also systematic reviews of teaching methods in the beginning of the project to inform this present study which will then be evaluated in randomized controlled trials in three countries in Africa.

 In order to enhance readability and help readers to understand the complexity of the whole project, authors reported details that had already been published elsewhere.

 This study is highly relevant and it was great to have access to the website. The manuscript is well written. However, authors should rather refer to already available publications and instead should provide further information on the following aspects:

1. The conducted *“Individual interviews employing user testing methods” *comprised three parts. Some teachers were interviewed right after a pilot lesson, so they had their experiences from the lesson fresh in mind. Please add information how many of them already taught a pilot lesson before the interview and whether these teachers also had access to the teachers guide.
2. Piloting and observation: The authors reported that early pilots involved single lessons. Were all lessons piloted as single lessons?
3.  The authors translated several of the audio recordings from individual and group interviews. Please add information on the selection of the recordings and why it was done.
4. The authors provide a teacher`s guide and also reported that lack of competences was a relevant issue. Please clarify whether participation in the workshop for teachers was optional or mandatory and whether teachers competences were assessed.

Is the work clearly and accurately presented and does it cite the current literature?

Yes

If applicable, is the statistical analysis and its interpretation appropriate?

Not applicable

Are all the source data underlying the results available to ensure full reproducibility?

Yes

Is the study design appropriate and is the work technically sound?

Yes

Are the conclusions drawn adequately supported by the results?

Yes

Are sufficient details of methods and analysis provided to allow replication by others?

Partly

Reviewer Expertise:

Critical health literacy, evidence-based health information, informed shared decision making

I confirm that I have read this submission and believe that I have an appropriate level of expertise to confirm that it is of an acceptable scientific standard, however I have significant reservations, as outlined above.

# Response

**Published:** 2024-03-06

**Authors:** Sarah Rosenbaum

**Affiliations:** Norwegian Institute of Public Health, Norway

**Response to Peer reviewer #1**

 Thank you very much for your time reviewing this article and your constructive questions. I am answering them below and updating the manuscript at the same time.

**Question 1: The conducted *“Individual interviews employing user testing methods” *comprised three parts. Some teachers were interviewed right after a pilot lesson, so they had their experiences from the lesson fresh in mind. Please add information how many of them already taught a pilot lesson before the interview and whether these teachers also had access to the teachers guide.**

*Reply 1a & b:*

**1a: How many taught a pilot lesson before the interview:** I’ve made changes to Table 6 so that this information is added there. I did this by rechecking with the PhD fellows about the participant data, and we found small errors in the original table, so these are also fixed.

**1b: Whether teachers had access to the teachers guide: **In the first development phase, we had not begun work on the teacher's guide, so no teachers had access to this in the early interviews or pilots. Regarding the second development phase, I’ve edited the text in the paragraph “Piloting and observation” so that the following information is provided: “In the final pilot, teachers taught all the lessons over a school term, with access to a complete prototype of the resources including the teacher’s guide.”

**Question 2: Piloting and observation: The authors reported that early pilots involved single lessons. Were all lessons piloted as single lessons?**

*Reply 2:*

 No. I have added the following clarifying text under “Piloting and observation”:

 “Initially we sought to try out different approaches to overarching questions that were not lesson specific (e.g. how much content, what kind of lesson structure, what kind of teaching strategies to build on, how to format the materials). As we made formatting and content decisions, we gradually developed a full set of lessons, not all of which were piloted individually in the the first development phase."

**Question 3: The authors translated several of the audio recordings from individual and group interviews. Please add information on the selection of the recordings and why it was done.**

*Reply 3:*

 I changed the text in the first paragraph under “Data analysis” to reflect more accurately what was done in each country: “In Uganda and Kenya, all interviews were carried out in English and transcribed. In Rwanda, interviews were carried out in Kinyarwanda and transcribed in English. There, the PhD fellow and research assistant relied on notes they made during interview sessions, and selected some recordings to review when further clarification of their notes was needed.”

**Question 4: The authors provide a teacher`s guide and also reported that lack of competences was a relevant issue. Please clarify whether participation in the workshop for teachers was optional or mandatory and whether teachers competences were assessed.**

*Reply:*

 The workshop was a part of the intervention that was tested in three randomised trials evaluating the effect of these resources, and reported in those studies. Participation in the workshop was not mandatory. Teachers’ competences were not assessed at the end of the workshop, but they were assessed in the trials at the end of all the lessons. You can find the trial results for both students and teachers in Table 3 of this article: Effects of the Informed Health Choices secondary school intervention: A prospective meta-analysis: https://onlinelibrary.wiley.com/doi/10.1111/jebm.12552

 I have added the following short summary of the results of this prospective meta-analysis in the Conclusions section, along with the corresponding citation reference: “A Prospective meta-analysis synthesizing the results of the three randomised trials has shown that the resources led to a large improvement in the ability of students and teachers to think critically about health choices, though only 42% of students achieved a passing score[64]. Next research steps are well underway: process evaluations of the three trials and one-year follow up studies.”

**Other updates to reflect referenced studies that are now published:**

- Reference #52 has been updated as this article has now been published.
- Reference #64 in the Conclusion is new, as the trial results are now published.
